# Dynamic regulatory processes among child welfare parents: Temporal associations between physiology and parenting behavior

**DOI:** 10.1017/S0954579423000949

**Published:** 2023-08-07

**Authors:** Xutong Zhang, Lisa M. Gatzke-Kopp, Elizabeth A. Skowron

**Affiliations:** 1 School of Psychology and Cognitive Science, East China Normal University, Shanghai, China; 2 Department of Psychiatry and Behavioural Neurosciences, McMaster University, Hamilton, ON, Canada; 3 Department of Human Development and Family Studies, The Pennsylvania State University, University Park, PA, USA; 4 Department of Psychology, University of Oregon, Eugene, OR, USA

**Keywords:** regulation, dynamic systems, parenting behaviors, physiology, child maltreatment

## Abstract

This study examined how temporal associations between parents’ physiological and behavioral responses may reflect underlying regulatory difficulties in at-risk parenting. Time-series data of cardiac indices (second-by-second estimates of inter-beat intervals – IBI, and respiratory sinus arrhythmia – RSA) and parenting behaviors were obtained from 204 child welfare-involved parents (88% mothers, *M*_age_ = 32.32 years) during child-led play with their 3- to 7-year-old children (45.1% female; *M*_age_ = 4.76 years). Known risk factors for maltreatment, including parents’ negative social cognitions, mental health symptoms, and inhibitory control problems, were examined as moderators of intra-individual physiology-behavior associations. Results of ordinary differential equations suggested increases in parents’ cardiac arousal at moments when they showed positive parenting behaviors. In turn, higher arousal was associated with momentary decreases in both positive and negative parenting behaviors. Individual differences in these dynamic processes were identified in association with parental risk factors. In contrast, no sample-wide RSA-behavior associations were evident, but a pattern of increased positive parenting at moments of parasympathetic withdrawal emerged among parents showing more total positive parenting behaviors. This study illustrated an innovative and ecologically-valid approach to examining regulatory patterns that may shape parenting in real-time and identified mechanisms that should be addressed in interventions.

## Introduction

Involvement with the child welfare system is often associated with risk factors including parenting difficulties, mental health symptoms, and socioeconomic adversity (Dubowitz et al., 2011; Stith et al., 2009). The goals of child welfare agencies when working with families include not only ensuring children’s safety, but also providing services to support or remedy caregiving processes and preventing negative impacts on children’s well-being (U.S. Department of Health & Human Services et al., 2022). Improving caregiving skills has been a central approach to preventing at-risk parenting and related developmental consequences for children (van der Put et al., 2018). In particular, many programs focus on parenting in early childhood, which is a critical period for the development of children’s self-regulation (Green et al., 2023) and when diverging trajectories of behavioral adjustment emerge (Fanti & Henrich, 2010). Positive changes in caregiving skills through parenting training have been shown to produce improvements in self-regulation among 3- to 6-year-old children exposed to early adversity (Speidel et al., 2020). This requires prevention programs to not only teach specific parenting skills but also address parents’ underlying difficulties that may give rise to negative parenting behaviors and/or interfere with positive parenting behaviors, so that the short-term parenting training can turn into sustainable gains in function (Sanders et al., 2019). However, studies on parents’ self-regulation or related difficulties often measure trait-like abilities (e.g., inhibitory control) or manifested psychopathological symptoms. Although these indicators explain individual differences in parenting, they are less informative on how practitioners can facilitate improvements within specific parenting contexts. This gap calls for work on how regulatory dynamics unfold during parent-child interaction.

Theoretical perspectives on the difficulties underlying at-risk parenting point to conflicts between parents’ internal states, particularly emotional responses to parenting demands, and their caregiving role. Parenting involves ebbs and flows of emotions (Dix, 1991), but when parents are unable to regulate emotional responses elicited by caregiving demands, their internal arousal may prompt harsh and controlling parenting behaviors or lead to parents disengaging from the interaction to cope with their distress (Skowron et al., 2010; Smith, 2003). To examine these processes empirically, researchers have sought to uncover real-time, dynamic relations between physiological indicators of parent emotional arousal and observed parenting behaviors, to shed light on potential “drivers” of at-risk parenting. The emerging evidence suggests that engaging in positive parenting behaviors that are typically experienced as enjoyable can be physiologically taxing for some at-risk parents; and when experiencing heightened arousal, they may have difficulty maintaining appropriate parenting and even engage in harsh and controlling behaviors as maladaptive regulatory attempts (e.g., Borelli et al., 2018; Skowron et al., 2013). However, those results have not been replicated on a continuous, moment-to-moment basis that is more closely aligned with how caregivers’ physiological and behavioral responses unfold during interactions.

Recent research in lower-risk families illustrated how parents’ physiological responses and child-directed behaviors continuously predict each other’s moment-to-moment changes may reflect their self-regulation (Zhang et al., 2022). These dynamic patterns demonstrated a balance between internal and external demands (i.e., momentary arousal predicted increases in responsive parenting behaviors, while responsive parenting predicted decreases in parent arousal), opposite to what we might expect in at-risk parenting. To advance our understanding of parents’ regulatory difficulties, the present study sought to replicate and extend previous findings using methods that capture how physiological responses may be temporally linked to changes in real-time parenting. In a new sample of child welfare-involved parents, we aimed to examine the dynamic associations between parents’ cardiac responding and observed parenting behaviors while in semi-structured play with their children. We also aimed to test how these dynamic physiology-behavior associations varied by parents’ psychosocial characteristics associated with the risk for child maltreatment.

### Regulatory difficulties and at-risk parenting

The biological and experiential transition to parenthood is typically accompanied by an increase in the salience of child-related cues, especially those that signal needs for attention and care, as well as enhanced responses in brain regions that promote approach motivations and cognitive functioning (Ferrey et al., 2016; Swain et al., 2014). These mechanisms are thought to help parents engage in caregiving behaviors while regulating their own emotions (Rutherford et al., 2015). Researchers have further conceptualized how parental self-regulation unfolds in the context of parenting, such that when external demands to attend to children’s needs create perturbations in parents’ internal states (e.g., momentary increases in physiological arousal), parents manage to engage in behaviors that optimally respond to such needs, which in real-time facilitate parents’ recovery from perturbations (Gatzke-Kopp et al., 2022; Zhang et al., 2022). However, there may be substantial individual differences in those processes. For example, among parents with regulatory difficulties, the patterns of physiological responses may be associated with dampened positive parenting behaviors and/or reinforced negative parenting behaviors. Identifying such patterns is critical for understanding the negative responses and lack of positive responses observed among at-risk parents even to normal child behaviors (e.g., Lunkenheimer et al., 2017; Wells et al., 2020).

The social information processing theories of child maltreatment posit that at-risk parents often hold hostile attributions toward children’s behaviors and unrealistic expectations about parenting experiences (Azar & Weinzierl, 2005; Milner, 2003). This may lead to negative emotional arousal not only in typically challenging parenting situations (e.g., child tantrums) but also in situations that are nondemanding or enjoyable for most parents (e.g., free play). For example, one study found that abusive mothers showed heightened physiological arousal to both infant cries and smiles, whereas non-abusive mothers showed increased arousal only to infant cries and stable or decreasing arousal to infant smiles (Frodi & Lamb, 1980). Another study found that when physically abusive mothers showed more positive parenting behaviors in a given 30-s segment of observation, their physiological arousal tended to increase in the same segment; and in a parallel model, higher maternal arousal predicted increases in subsequent hostile parenting (Skowron et al., 2013). These findings suggest that for some parents, even positive child-related stimuli and typical parenting tasks may be experienced as demanding and physiologically taxing. Furthermore, a recent meta-analysis confirmed that parents at-risk for child maltreatment often have more difficulties regulating their emotions to support goal-directed behaviors (Lavi et al., 2021). For these parents, when the interaction with children becomes demanding or distressing, heightened arousal may more easily interfere with appropriate parenting behaviors and prompt harsh or avoidant responses.

To summarize, contrary to the dynamic patterns of physiology and behavior observed in low-risk samples that reflect parents’ ability to both be responsive to their children and restore their own emotional equilibrium (Zhang et al., 2022), parents at-risk for maltreatment may experience regulatory difficulties that manifest as conflicts between internal and external demands (Skowron et al., 2013; Wells et al., 2020). Parenting efforts that are supportive for the child may be experienced as demanding, leading to perturbations to the physiological system. Meanwhile, heightened arousal may have a negative impact on parenting behavior, devolving into feedback loops in which positive parenting drives arousal, and arousal presents as a risk factor for decreased positive and increased negative behaviors. If such underlying mechanisms exist, corresponding temporal associations should be evident that link dynamic changes in parents’ internal physiological responses to their *real-time* parenting behaviors.

### Regulatory processes reflected in physiology-behavior dynamic associations

To understand parents’ regulatory processes that shape their parenting behaviors on a moment-to-moment basis, ambulatory measures of physiology provide a powerful tool. Physiological reactivity indexed by changes in the autonomic nervous system (ANS) is a component of the organized responses that constitute emotional arousal, capturing how the organism is preparing to engage with the ongoing circumstance (Levenson, 2014). Common measures of ANS responses, such as cardiac reactivity, can be taken continuously and unobtrusively during parent-child interaction. These allow researchers to align the time-series data on parents’ physiological responses and parenting behaviors, and to examine the moment-to-moment associations that may reflect dynamic processes of regulation during parenting.

Two cardiac indices that are closely associated with emotional arousal, and may capture internal perturbations associated with behavioral responses, are inter-beat intervals (IBI) and respiratory sinus arrhythmia (RSA; Kreibig, 2010). IBI refers to the distance between consecutive heartbeats. It is an indicator of overall cardiac arousal (shorter IBI indicates greater arousal), shaped by both the sympathetic and the parasympathetic branches of the ANS. These two branches can form diverse patterns of coordination, which vary across individuals and specific contexts, to meet situational demands (Gatzke-Kopp & Ram, 2018). Changes in IBI thus capture integrated autonomic cardiac control with fine temporal resolution, reflecting how demanding the ongoing circumstance is for an individual’s physiological system. Although there has not been evidence on its temporal associations with behaviors in the context of parenting, dynamic changes in IBI on the timescale of seconds or milliseconds have been linked to the coordination of behaviors and reactivity to others’ emotion cues in social contexts (Feldman et al., 2011; Oliveira-Silva & Gonçalves, 2011).

In addition to overall cardiac arousal, parasympathetic regulation (i.e., withdrawal and reinstatement of inhibition over-arousal) is theorized to play a central role in the moment-to-moment adjustment of physiology to support flexible emotional and behavioral responses that are central to social functioning (Porges, 2007; Smith et al., 2017). Parasympathetic activity is usually measured by RSA, which captures heart rate variability within the respiratory frequency range (higher RSA reflects greater parasympathetic inhibition over cardiac arousal; Berntson et al., 1993). Consistent with the theoretical perspectives, estimates of second-by-second RSA are reactive to dynamics changes in emotional stimuli (Ravindran et al., 2021), and a pattern of RSA decreases during stressful social interaction followed by timely recovery to higher levels has been related to better psychosocial functioning in both child and adult samples (Miller et al., 2013; Shahrestani et al., 2014; Shahrestani et al., 2015). However, some studies suggested that maintaining higher levels of RSA when facing social challenges may be related to efforts to self-regulate and less *real-time* consumption of cognitive resources by stress (Butler et al., 2006; Roos et al., 2017). In parental samples, findings on the implications of RSA reactivity for parenting are mixed, although a recent study suggested that variations in the direction and degree of dynamic associations between RSA and real-time parenting may reflect self-regulation efficiency or patterns (Zhang et al., 2022). That is, when parenting demands elicit momentary parasympathetic withdrawal, some parents may manage to maintain or increase positive engagement whereas others show compromised parenting quality.

These two cardiac indices, one reflecting overall cardiac arousal and the other reflecting parasympathetic inputs, can both support analyses of physiology-behavior associations on a moment-to-moment basis. RSA has been examined in terms of its temporal association with parenting behaviors, both on a second-by-second timescale (Zhang et al., 2022) and across 30-s segments (Skowron et al., 2013), and in the latter study linked to types of maltreatment. Meanwhile, greater overall cardiac arousal in response to child-related stimuli (averaged across minutes) has been related to maltreatment or the potential for abusive behaviors (Frodi & Lamb, 1980; McCanne & Hagstrom, 1996), but its associations with real-time, dynamically changing behaviors among parents have not been explored. Based on the conceptual hypotheses of regulatory difficulties in at-risk parenting, we aimed to examine both IBI and RSA in association with real-time parenting behaviors among child welfare parents.

### Inter-individual differences in parents’ regulatory processes

In addition to examining the prototypical physiology-behavior associations in the sample, another aim of this study was to understand how these dynamic processes may vary across parents in association with well-recognized risk factors for child maltreatment. According to the social information processing model, one set of major risk factors for problem parenting includes negative social cognitions, especially those related to the child or parenting demands (Azar & Weinzierl, 2005; Milner, 2003). Abusive and neglectful parents tend to report more harsh attributions of child behaviors as well as greater subjective stress related to parenting (Stith et al., 2009). In addition to survey measures, laboratory-based measures of deficits in processing facial emotions (e.g., perceiving ambiguous cues as negative) have been observed among both abusive and neglectful parents (Francis & Wolfe, 2008; Camilo et al., 2021), and lower accuracy in identifying child positive facial expressions was found among neglectful mothers (Hildyard & Wolfe, 2007). Therefore, we hypothesized that parents who display subjective perceptions of and/or objectively measured negative social cognitive processes may experience their interactions with their children as more demanding. This may manifest as increased physiological arousal when engaging in appropriate and supportive parenting behaviors.

We also sought to examine whether parental risk in the form of mental health symptoms and inhibitory control problems would be linked to individual differences in the dynamic associations between physiology and parenting behaviors. We reasoned that these risk factors may be related to lower abilities to engage in goal-directed actions, especially under emotional demands. This may thus make it difficult for parents to sustain positive parenting behaviors and not act on negative parenting behaviors when they experience heightened arousal. For example, mothers with more depressive symptoms show steeper increases in negative expressions toward their children as the aversiveness of child behaviors increases (Dix et al., 2014), and highly anxious mothers may engage in controlling parenting behaviors to regulate their own cardiac arousal (Borelli et al., 2018). Yet another body of research has found a lack of flexible parasympathetic engagement during social interactions in association with the risk for psychopathology (McKillop & Connell, 2018; Shahrestani et al., 2015), pointing to the possibility that RSA-behavior dynamic associations may be less evident among parents with more mental health symptoms. Among non-parental adults, inhibitory control performances can be compromised by stress especially when there are greater demands on the physiological system (Roos et al., 2017). Through the lens of dynamic regulatory processes, we aimed to examine whether similar regulatory difficulties would manifest as an increase in negative parenting behaviors at moments of cardiac arousal or parasympathetic withdrawal, and whether such association would be stronger among parents with inhibitory control problems.

### The present study

The present study aimed to address two questions regarding the regulatory processes unfolding in the context of parenting among child welfare-involved parents. First, we aimed to examine whether the dynamic associations between parents’ physiology and observed behaviors would show patterns reflecting regulatory difficulties in sustaining positive parenting behaviors and containing negative parenting behaviors. We hypothesized that when interacting with their children, parents’ physiological arousal would increase (i.e., shortening IBI and decreasing RSA) when they utilized positive parenting behaviors that require child-centered efforts (e.g., praising children’s appropriate behaviors, responding verbally in a reciprocal way). On the contrary, engaging in negative parenting behaviors may not predict increases in arousal, and may even predict a decrease in arousal, consistent with an at-risk pattern of using harsh or controlling actions to regulate parents’ own emotional reactivity. Meanwhile, we hypothesized that higher arousal would predict decreases in positive parenting behaviors and, in line with past research, increases in negative parenting behaviors. It should be noted that physiology and behavior can be influenced by many factors beyond those accounted for in this study. We did not assume that the dynamics would always unfold as a three-part sequence, where only heightened arousal following positive parenting behaviors would predict decreases in parenting quality. Rather, we theorized that among at-risk parents, positive parenting behaviors would contribute to heightened physiological arousal, which in itself may present as a risk factor for decreased parenting quality, and then tested whether the dynamic associations between pairs of variables (see Data Analysis) showed patterns consistent with the conceptual hypotheses.

Second, we sought to examine whether the physiology-behavior dynamic associations in the context of parenting were moderated by parent characteristics relevant to maltreatment risks, including indicators of negative social cognitive processes (i.e., harsh child attributions, child-focused parenting stress, and patterns of response to emotion cues), mental health symptoms, and problems with inhibitory control. As reviewed in the earlier section, we tested whether negative social cognitions would be associated with greater increases in cardiac arousal and/or greater parasympathetic withdrawal when engaging in appropriate parenting efforts (i.e., consistent with experiencing parenting as more demanding and physiologically taxing). We also hypothesized that greater mental health symptoms and inhibitory control problems would be associated with difficulties in regulating behavioral responses in emotionally demanding situations. This would manifest as decreased positive parenting and increased negative parenting at moments of greater cardiac arousal and/or parasympathetic withdrawal. We also examined the hypothesis that mental health symptoms may be related to a lack of flexible parasympathetic engagement and thus less evident RSA-behavior associations across the board. Examining these risk factors for child maltreatment as potential moderators would help connect the analyses of the novel intra-individual dynamics with the broader literature on at-risk parenting.

## Method

### Participants

Data were drawn from a larger project examining the efficacy of a parenting intervention program in the child welfare context. The present study used data collected from families before they were randomized to different conditions or received any intervention related to the project. Participants were recruited through the Department of Human Services (DHS) from April 2016 to June 2019. Eligibility criteria at the time of enrollment included (1) the parent was at least 18 years old and the participating child’s biological parent or custodial caregiver, (2) the child was between 3 and 7 years old, (3) the parent had no prior history of perpetrating child sexual abuse, and (4) the parent provided written informed consent to participate for themselves and their child. Further details about recruitment and sample size determination are available in the protocols preregistered at clinicaltrials.gov (NCT02684903).

The sample included 204 parents (180 mothers and 24 fathers; *M*_age_ = 32.32 years, *SD* = 6.38, ranging from 18 to 64 years) and their children between 3 and 7 years of age (45.1% female; *M*_age_ = 4.76 years, *SD* = 1.40). The majority of parents (98.0%) were the biological parents of the children. Most parents identified as European American/White (70.1%), and the rest identified as Hispanic American/Latina (2.5%), African American/Black (2.0%), Asian/Pacific Islanders (1.5%), Native American/Alaskan Aleut (1.5%), Multiracial/Multiethnic (20.6%), or did not report their race/ethnicity (0.5%). About a quarter of parents were married or living with a partner (28.4%), and the others were separated (10.3%), divorced (10.8%), single (45.6%), or in another relationship status (4.9%). Half of the sample held a high school diploma as their highest degree (49.5%), 16.7% did not finish high school, and 33.8% completed vocational training or held an associate or bachelor’s degree. More than half of the parents were not employed at the time of the study (53.4%). The annual household income was relatively low in this sample (*M* = $18,582, *SD* = $13,364, 10 and 90% percentiles = $6,000 and $36,000; 19.1% of the parents did not report their income), and 83.8% were receiving food stamps. Based on the families’ child welfare records coded using the Maltreatment Classification System (Barnett et al., 1993), 41.7% of the dyads (*n* = 85) had been involved in reports of child maltreatment before this study. Most previous reports were related to physical neglect (*n* = 64) with the rest categorized as “other types.”

### Procedures

Enrolled families were invited to a laboratory assessment before they were assigned to any intervention related to the project. After obtaining written informed consents from parents, and from a family’s caseworker if DHS Child Welfare maintained legal custody, trained research assistants applied noninvasive electrodes on each member of the dyad, which was connected to a wireless device that recorded cardiac signals. Dyads were given time to get comfortable with the devices and then participated in a series of dyadic and individual parent or child tasks. Parents also completed surveys on demographics, parents’ and children’s functioning, and environmental risk exposures. To accommodate the wide variability in parent literacy, an interview format was used for the survey and the trained research assistant entered parents’ answers. The present study focused on a 5-min *child-led play* task as part of the standard Dyadic Parent-Child Interaction Coding System tasks (DPICS; Eyberg & Funderburk, 2011). The dyads were provided with a standardized set of toys, and parents were instructed to let their children decide what to play and to follow their children’s lead. The task did not impose other external challenges on the dyad and was administered before the other two DPICS tasks that asked parents to lead or direct the child. All tasks were video-recorded for transcription and offline observational coding. The families received compensation for attending the assessment and for transportation costs. Refreshments, rest breaks, and childcare were provided during the assessment, and the children received a small prize. All procedures were approved by the Institutional Review Boards of the University of Oregon (IRB #07102014) and the Oregon Department of Human Services (#188).

### Measures

#### Physiological and behavioral dynamics during child-led play

##### IBI and RSA.

Parents’ electrocardiography (ECG) data were collected during the child-led play task using the Mindware ambulatory device (MND-50–2303-00; Mindware Technologies LTD., Westerville, OH). Signals were recorded through three electrodes (placed on participants’ right collar bone, lower left rib, and lower right rib) at a sampling frequency of 500 Hz and were transmitted wirelessly to the Mindware BioLab software (version 2.4). ECG data were preprocessed offline in the Mindware HRV software (version 3.1.3), which identified R peaks algorithmically. Trained research assistants visually inspected the ECG data and manually corrected erroneously identified or missing R peaks when waveforms were clear. Cleaned IBI series were then output in 30-s segments. If a segment contained lost or corrupted signals such that R peaks could not be identified, that segment was not output; in those cases, a 30-s value was inserted into the IBI series to ensure its alignment with the actual flow of time, which was handled as missing data in later processing.

The output from Mindware was then imported into R (R Core Team, 2016). For each participant, the IBI series from individual segments were compiled into one consecutive IBI series for the entire task. Second-by-second estimates of IBI and RSA were then calculated using the RHRV package (Martínez et al., 2017). First, the input IBI series were filtered for outliers based on a preset possible range of IBI values (0.2–2 s) and the algorithm of the FilterNIHR function in RHRV (Martínez et al., 2017; Vila et al., 1997). Outliers were removed, while a separate variable tracked the accumulated time in the original IBI series so that the removal of outliers would not disrupt the temporal alignment of data. Second, from the filtered IBI data, second-by-second estimates of equidistant IBI series were generated using cubic spline interpolation (sampling frequency = 1 Hz). Third, for the calculation of RSA, a separate equidistant IBI series was generated with the same interpolation method but a 4 Hz sampling frequency to adequately capture the variability in uninterpolated IBI series. Second-by-Second RSA estimates were calculated using overlapping 30-s windows that each moved forward 1 s through the equidistant IBI series. IBI data within each window was subject to a Hamming window function that up-weights the center of the window, and a short-time Fourier transform was applied to obtain an estimate of the power spectrum assigned to represent the 15^th^ second of the window. Based on the power spectrum estimates, second-by-second RSA was then computed as the natural log of power within the adult respiration frequency band (0.12–0.40 Hz; Berntson et al., 2007). This approach of obtaining second-by-second RSA estimates has been shown to effectively capture dynamic changes consistent with shifts in emotional stimuli (Ravindran et al., 2021). Because this method requires 30-s of continuous data to compute the RSA estimate for the 15th second in a given window, RSA estimates were not available for 14 and 15 s respectively at the beginning and the end of the task. Additionally, when there was a segment of missing data in the IBI series, RSA values would be missing from 15 s before the segment until 14 s after the segment.

ECG data were missing completely for two parents due to file error or that the parent had a pacemaker so did not wear the electrodes. Across the rest of the sample, 1.6% of second-by-second IBI values were missing and 9.9% of second-by-second RSA values (including the missing data at the beginning and end of the task) were missing during this task. To put IBI and RSA values on similar scales, this paper used seconds instead of milliseconds as the unit of IBI. The task-average values of second-by-second IBI ranged from 0.54 to 1.24 s across parents, *M* (*SD*) = 0.76 (0.12), and RSA ranged from 2.10 to 8.93, *M* (*SD*) = 5.36 (1.39).

##### Positive and negative parenting behaviors.

Video recordings of the child-led play task were transcribed and observationally coded using the well-validated Dyadic Parent-Child Interaction Coding System-IV (DPICS-IV; Eyberg et al., 2013; Nelson & Olsen, 2018). *Positive parenting behaviors* (i.e., PRIDE skills) captured parents’ verbal expression of warmth, attentiveness, and responsiveness in this context, and included labeled and unlabeled Praise (e.g., “I love how gentle you are being with the toy cars.”; “You got them all right!”), Reflections (restating all or part of the child’s verbalizations, e.g., when the child states, “my truck is red”, the parent responds, “your truck IS red”), and behavior Descriptions (e.g., when the child picks a crayon, the parent says, “You’ve chosen a purple crayon!”). Note that Imitation and Enjoyment behaviors are not coded in the DPICS-IV. In this task where parents were tasked with following the child’s lead, negative parenting behaviors refer to verbalizations that convey negativity or interference with autonomy, labeled “Don’t skills”, that is, commands (e.g., “Stop yelling!”, “Hurry up!”), questions (e.g., “Why are you using that red crayon?”), and criticism/negative talk (e.g., “That looks terrible!”).

Trained research assistants conducted event-based coding of each category of behaviors in Noldus Observer XT (Zimmerman et al., 2009). For example, they identified every parental utterance consistent with the definition of labeled praise in the video, and the software automatically recorded the time stamp of the initiation of each utterance. The output from Noldus was then imported into R and transformed into time-series data of whether a coding of positive or negative parenting behaviors was present (1) or not (0) for each second. Observational data were available for all but one family (due to file errors). The total frequency of parenting behaviors during the child-led play task ranged from 0 to 27 for positive parenting behaviors, *M* (*SD*) = 5.27 (4.44), and from 0 to 67 for negative parenting behaviors, *M* (*SD*) = 23.16 (11.55). Most parents (93%) showed at least one instance of both positive and negative parenting behaviors during child-led play.

**Figure 1.**
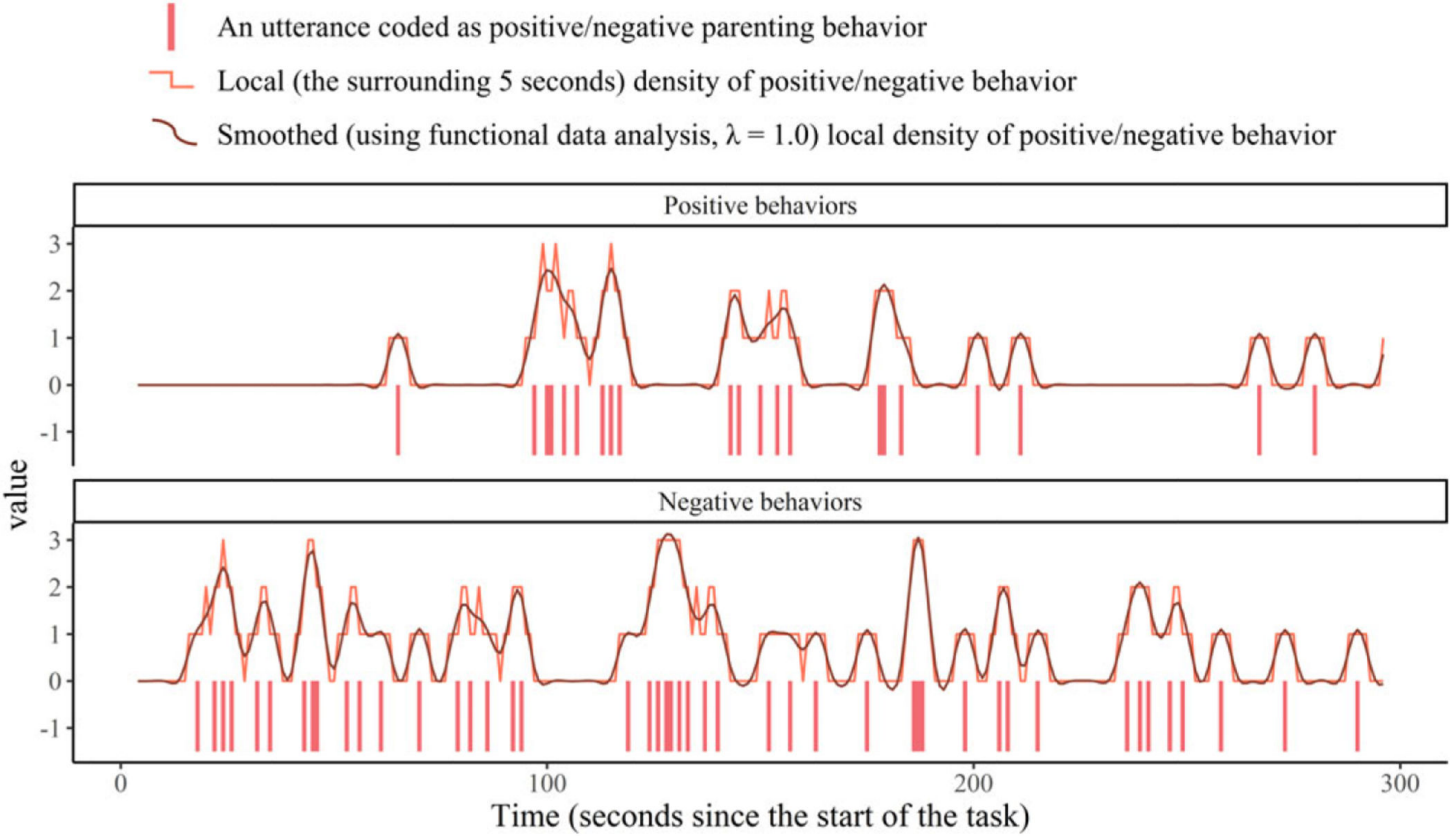
Visualization of one parent’s positive and negative parenting behaviors and the time-series estimates of their local density during the child-led play task.

To better represent the temporal variability in parents’ positive or negative behaviors, we computed their second-by-second local density using a 5-s moving window. For example, the local density of positive parenting behaviors for a given second is the frequency of positive behaviors in the 5-s window centering this second. As illustrated in Figure 1, higher local density indicates a more frequent presence of positive or negative skills surrounding the moment.

#### Proposed moderators: risk factors for child maltreatment

##### Harsh child attributions.

Parents completed the Structural Analysis of Social Behavior Intrex Questionnaires – Short Form, a self-report assessment of intra- and inter-personal perceptions and characteristics (Benjamin et al., 2006). Two items from the Child with Me – Transitive Scale (Clusters 15 – child controls the parent, and 16 – child is harsh and critical toward parent) were used to measure parental perceptions of their children’s behaviors toward them as controlling or hostile. Each item was rated on a continuous scale ranging from 0 (does not apply at all/never) to 100 (applies perfectly/all the time). Parents’ perceptions of their children as being in control of their interaction or holding hostile intentions, although not fully overlapping (*r* = .22, *p* = .002 in this sample), both represent negative child attributions and contribute to the risk of maltreatment (Azar & Weinzierl, 2005; Rodriguez & Richardson, 2007). Therefore, a composite score of harsh attribution was calculated by summing the two items. Scores varied across the full range (0–200) in this sample, *M* (*SD*) = 71.19 (45.66), with higher scores reflecting perceptions of greater child hostile control toward parent.

##### Child-focused parenting stress.

Parents responded to 24 items from two subscales of the Parenting Stress Index – Short Form (PSI-SF; Abidin, 2012). The Difficult Child subscale measures the extent to which parents perceive their children as difficult to manage or care for (e.g., “My child does a few things that bother me a great deal.”). The Parent-Child Dysfunctional Interaction subscale captures parents’ dissatisfaction with their child and the interaction between them and a lack of bonding (e.g., “My child smiles at me much less than I expected.”). Only these two subscales were administered given a focus on at-risk parents’ experience of stress specifically related to perceptions of the child or parent-child relationship. Each item was rated on a 5-point Likert scale from 1 (strongly disagree) to 5 (strongly agree). For easy interpretation, the percentile score of each subscale was used in this study, indexing the relative standing of parents’ scores among all parents that were assessed during the development and testing of PSI-SF. Furthermore, because the two subscales overlap conceptually and their scores were highly correlated in this sample (*r* = .62, *p* < .001), a composite score of *child-focused parenting stress* was calculated by averaging the two subscale percentiles. Higher scores represent greater parenting stress related to the perception of the child and parent-child interaction. The composite percentile score ranged from 3 to 99 in this sample, *M* (*SD*) = 64.65 (24.75).

##### Response to emotion cues.

Parents completed an Emotional Go/No-Go task (Schulz et al., 2007), based on which a range of behavioral performance indicators related to the processing of and response to emotions can be obtained (Tottenham et al., 2011). Parents were presented with images of neutral, angry, happy, sad, and fearful adult faces on a laptop, and were instructed to press a response key when they saw a target emotion and refrain from pressing the key when they saw a nontarget emotion. A total of eight blocks were run, including four Neutral-target blocks (pressing the key when seeing neutral faces) and four Emotion-target blocks with angry, happy, sad, and fearful faces as targets (target designation counterbalanced across blocks of trials). Given the interest in parents’ responses to positive and threat-related emotion cues, two focal indicators were examined. First, we examined the Hit Rate in the Happy-target block, that is, the rate of correctly pressing the key when a happy face was presented with neutral distractors. Second, we examined the False Alarm Rate toward Angry-distractors, that is, the rate of incorrectly pressing the key when an angry face (i.e., ‘No-Go’ trials in the Neutral-target, Anger-distractor block) was presented. However, the distribution of those two proportion scores (possible range = 0 to 1) was either negatively skewed (Hit Rate to happy faces, *M* = 0.72, *SD* = 0.21, median = 0.80) or zero-inflated (False Alarm Rate to angry faces, *M* = 0.06, *SD* = 0.11, 59.8% of parents had no false alarm response). Therefore, the two scores were dichotomized, such that the accurate *hit rate for positive emotion* represented whether parents’ accuracy in identifying and responding to Happy-target faces was higher than the sample median (1) or not (0), and *false alarm rate to anger* represented whether parents displayed any false alarm to Angry-distractor (1) or not (0). We also included parents’ average reaction times when correctly distinguishing the target from the distractor as covariates (Happy-target block reaction times ranged from 0.32 to 0.49 s, *M* = 0.41, *SD* = 0.03; Neutral-target/Angry-distractor block reaction times ranged from 0.33 to 0.51 s, *M* = 0.43, *SD* = 0.03; shorter reaction times indicated faster responses). Missing data (<4%) were due to parents not completing the Go/No-Go task (e.g., time constraints during the visit). See Table 1 for descriptive statistics.

##### Mental health symptoms.

Parents’ depressive and anxiety symptoms were assessed through self-report using subscales of the Brief Symptoms Inventory, which has shown reliability and validity in measuring mental health problems (Derogatis, 2001; Rath & Fox, 2017). The Depression subscale includes 5 items capturing dysphoric moods and a lack of motivation and interest, and the Anxiety subscale includes 6 items capturing feelings of tension, nervousness, and terror. Each item was rated on a 5-point Likert scale from 0 (not at all) to 4 (extremely), and standardized T-scores were examined in this study (Depression T-scores ranged from 42 to 80, *M* = 55.86, *SD* = 9.36; Anxiety T-scores ranged from 38 to 80, *M* = 55.79, *SD* = 10.95 in this sample). Although depressive and anxiety symptoms may contribute to regulatory difficulties in different ways, their scores were highly correlated (*r* = .65, *p* < .001) and showed consistent patterns in association with the dynamic regulatory processes examined in this study. Therefore, the two T-scores were averaged into a composite score of mental health symptoms, which ranged from 40 to 80 in this sample (*M* = 55.83, *SD* = 9.24).

##### Inhibitory control problems.

Parents’ self-reported inhibitory control problems were assessed using the Inhibit Problems subscale in the Behavior Rating Inventory of Executive Function-Adult Version (BRIEF-A; Roth et al., 2005). This measure has shown adequate reliability and validity in assessing deficits in executive functioning among adults (Roth et al., 2014). The Inhibit Problems subscale contains 8 items regarding the control of impulses and the ability to inhibit attention or behaviors as needed. Parents indicated how often the action or ability described in each item was a problem for them on a 3-point scale (1 = never, 2 = sometimes, 3 = always a problem). Parents’ Inhibit Problems T-scores (*M* = 50, SD = 10) are based on norms drawn from community samples of U.S. adults (Roth et al., 2014), and ranged from 37 to 82 in the current sample, *M* (*SD*) = 55.60 (10.05), with higher scores indicating more inhibitory control problems.

### Data analysis

This study aimed to model the intra-individual temporal associations between parents’ physiological responses and observed parenting behaviors, as well as the inter-individual differences in those dynamic processes as related to parent characteristics. We applied ordinary differential equations (ODE) within a multilevel modeling framework, which can accommodate the nested nature of the data and has been applied in studies of dynamic regulation processes (e.g., Cole et al., 2017; Steele & Ferrer, 2011). ODE treats time as a continuous index, assuming that the dynamic processes are continuous even though indicators may be assessed at regular intervals. Compared to discrete-time modeling that imposes equally split time intervals, continuous-time modeling is more flexible in accounting for the timing of changes that may not unfold on a unified time course (de Haan-Rietdijk et al., 2017). We adopted pairs of first-order differential equations to examine how the levels of parental physiology and parenting behavior predicted each other’s momentary changes. The momentary change of each variable is quantified as smoothed derivatives, thus incorporating information on where it is heading toward the next moment and capturing temporal dynamics.

A two-step approach was adopted (Chow, 2019). Using the “getdx()” function in the *dynr* R package (version 0.1.16–27; Ou et al., 2019; functions from the *fda* R package were also incorporated; Ramsay et al., 2009), we used functional data analysis (FDA; Ramsay & Silverman, 2005) to obtain smoothed time-series estimates and first derivatives of IBI, RSA, and the local density of positive and negative parenting behaviors. To examine momentary changes in the variables (represented by first derivatives, indicating its direction and rate of change at a given moment), the time-series data were approximated using 5^th^ order B-splines functions with roughness penalty (penalizing the integrated squared 3^rd^ derivative). Guided by the generalized cross-validation index and visualizations of time-series dynamics, the smoothing parameter λ was set at 0.5 for IBI, 0.1 for RSA, and 1 for parenting behaviors (see Figure 1 and Figure S1 in the Supplementary Materials). For example, the smoothed level and change (first derivative) of IBI for individual *i* at time *t* are written as $IBI_{i}(t)$ and $\frac{dIBI_{i}(t)}{dt}$. The smoothed derivatives incorporate information from surrounding moments (temporally closer observations get greater weight), so a temporal predictive element was embedded to reflect how the variables are changing dynamically.

The hypothesized intra-individual physiology and behavioral processes were then modeled using four pairs of ODEs at the intra-individual level (level 1) of the multilevel models. For example, the first pair of ODEs were specified as:

(1) $$\frac{dIBI_{i}(t)}{dt}=a_{1i}+b_{1i}\left(IBI_{i}(t)-\bar{IBI_{i}}\right)+c_{1i}POS_{i}(t)+u_{1i}(t)$$

(2) $$\frac{dPOS_{i}(t)}{dt}=d_{1i}+e_{1i}{\mathrm{POS}}_{i}(t)+f_{1i}\left(IBI_{i}(t)-\bar{IBI_{i}}\right)+v_{1i}(t)$$

The momentary change of parental IBI for individual *i* at time $t(\frac{dIBI_{i}(t)}{dt})$ was modeled as a function of the intercept $\left(a_{1i}\right)$, the concurrent level of IBI relative to the person’s average ${\bar{IBI}}_{i}(b_{1i})$ and the local density of positive behaviors $\left(c_{1i}\right)$, as well as the residual $u_{1i}\left(t\right)$. The momentary change in the local density of positive behaviors for individual *i* at time $t(\frac{dPOS_{i}(t)}{dt})$ was modeled as a function of the intercept $\left(d_{1i}\right)$, the concurrent local density $\left(e_{1i}\right)$, concurrent IBI level $\left(f_{1i}\right)$, and the residual $v_{1i}\left(t\right)$. The parameters that account for variables’ intrinsic dynamics (e.g., $b_{1i}$ and $e_{1i}$) represent how their momentary levels relative to “set points” (task-average IBI and no observed behavior) predict the variables’ own momentary changes. For example, a negative *b*_1*i*_ would suggest that cardiac arousal tends to return to the individual’s task-average level in the course of dynamic changes (i.e., increasing IBI when lower than task average, and decreasing IBI when higher than task average). The remaining three pairs of ODEs examined the temporal associations between IBI and negative parenting ($a_{2i}$ to $f_{2i}$ ), between RSA and positive parenting ($a_{3i}$ to $f_{3i}$), and between RSA and negative parenting behaviors ($a_{4i}$ to $f_{4i}$), respectively. In the initial models, no additional predictor was entered at the inter-individual level (level 2), so the level 1 parameters were only modeled as a function of intercepts (representing estimates of the parameters for a prototypical parent in this sample) and random effects (representing inter-individual variability in the parameters), e.g., $a_{1i}=\gamma_{a10}+w_{a1i};b_{1i}=\gamma_{b10};c_{1i}=\gamma_{c10}+w_{c1i}$. Note that random effects were only estimated for the intercepts at level 1 and the parameters representing dynamic associations between physiology and behavior (i.e.,$w_{a1i}\text{to}w_{a4i},w_{c1i}\text{to}w_{c4i},w_{d1i}\text{to}w_{d4i},w_{f1i}\text{to}w_{f4i}$). The main hypotheses were tested by examining how IBI/RSA and parenting behaviors were related to each other’s momentary changes for the prototypical mother in this sample, represented by $\gamma_{c10}$ to $\gamma_{c40}$ and $\gamma_{f10}$ to $\gamma_{f40}$.

Next, parent characteristics were added as level 2 moderators of the intra-individual parameters of interest ($c_{1i}$to $c_{4i},f_{1i}$ to $f_{4i}$) to explain how they moderated the dynamic associations between physiology and behavior. The moderators (i.e., harsh child attributions, child-focused parenting stress, responses to emotion cues, mental health symptoms, and inhibitory control problems) were tested in separate models, although in the models of responses to emotional cues, the reaction time of the corresponding block was included in the same model. All predictors were standardized using sample means and standard deviations. The task-total frequency of positive or negative parenting behaviors was examined as a predictor in a separate model but also included as a covariate in the models involving the corresponding type of behavioral dynamics. For example, to examine how harsh attributions were associated with inter-individual differences in $c_{1i}$. equation (1) was expanded as

$$\frac{\mathrm{dIB}I_{i}(t)}{\mathrm{dt}}=(\gamma_{a10}+\gamma_{a11}\mathrm{Harsh}\;\mathrm{Attribution}\_{\mathrm{std}}_{i}\;\;+\gamma_{a12}\;\mathrm{Task}\;\mathrm{Average}\;\mathrm{Positive}\;\mathrm{Behavior}\_{\mathrm{std}}_{i}+w_{a1i})\;\;+\gamma_{b10}{\left({\mathrm{IBI}}_{i}(t)-\bar{{\mathrm{IBI}}_{i}}\right)}_{i}\;\;+(\gamma_{c10}+\gamma_{c11}\;\mathrm{Harsh}\;\mathrm{Attribution}\_{\mathrm{std}}_{i}\;\;+\gamma_{c12}\mathrm{Task}\;\mathrm{Average}\;\mathrm{Positive}\;\mathrm{Behavior}\_{\mathrm{std}}_{i}\text{+}w_{c\text{1}i}){\mathrm{POS}}_{i}(t)\;\;\text{+}u_{i}\text{(}t\text{)}$$

where the main effect of harsh child attributions on the change in parental IBI was estimated as $\gamma_{a11}$, and the interaction with the local density of positive parenting behaviors (i.e., harsh attributions moderating how the momentary density of positive parenting behaviors predicted changes in IBI) was estimated as $\gamma_{c11}$. Significant interaction effects were probed to estimate the intra-individual associations (e.g., *c*_1_, *f*_1_) at lower (one standard deviation below mean; *M* – *SD*) and higher (*M* + *SD*) levels of the risk factor.

None of the risk factor measures or task-average IBI and RSA scores differed by parent or child gender; none was correlated with child age either. The task-total frequency of positive or negative parenting behaviors differed by child but not by parent gender. Parents of boys showed more instances of both positive (*t* (201) = 1.98, *p* = .05) and negative (*t* (198) = 2.62, *p* = .01) behaviors across the task. Additionally, parents showed less positive behaviors with older children (see Table 1). However, because the total frequency of positive or negative parenting behaviors was already controlled for in all models examining inter-individual differences, we did not further include the basic demographic variables as covariates. We also considered respiration rate as a potential covariate. However, preliminary analyses showed that parents’ respiration rate during the child-led play task did not moderate the dynamic associations of RSA or IBI with parenting behaviors (for the moderating effects, *p*s = .22–.96) and thus would not impact the interpretation of findings at the intra-individual level. Furthermore, parents’ respiration rate was not significantly correlated with their frequency of utterance during the task (r=−.11,p=.12), total counts of positive and negative parenting behaviors, or any other parent characteristics measured (*r*s = −.08 to .07, *p*s > .26). Therefore, respiration rate was not included as a covariate in the final models.

All models were fit to the 59,762 repeated measures nested within 204 parents using the *nlme* package (version 3.1–153; Pinheiro et al., 2017) in R, with restricted maximum likelihood estimation and with incomplete data treated based on standard missing-at-random assumptions. Statistical significance was evaluated at α = 0.05.

## Results

As shown in Table 1, parents’ task-average IBI and RSA estimates were not correlated with the total frequency of positive or negative parenting behaviors during child-led play. Nor were task-average physiology or behavior frequency correlated with the parental risk factors for maltreatment. Child-focused parenting stress, mental health symptoms, and inhibitory control problems were positively correlated, but not correlated with harsh child attributions. Notably, harsh child attributions, child-focused parenting stress, and responses to emotion cues were not correlated, suggesting that they each captured unique aspects of social cognitive processes. In the Go/No-Go task, parents who showed longer reaction times (i.e., slower responses) on correct hit trials showed fewer false alarms to anger and lower accuracy in identifying positive faces. However, false alarm rates of anger and accuracy in identifying positive faces were not correlated with each other.

### Intra-individual regulatory processes during child-led play

**Figure 2.**
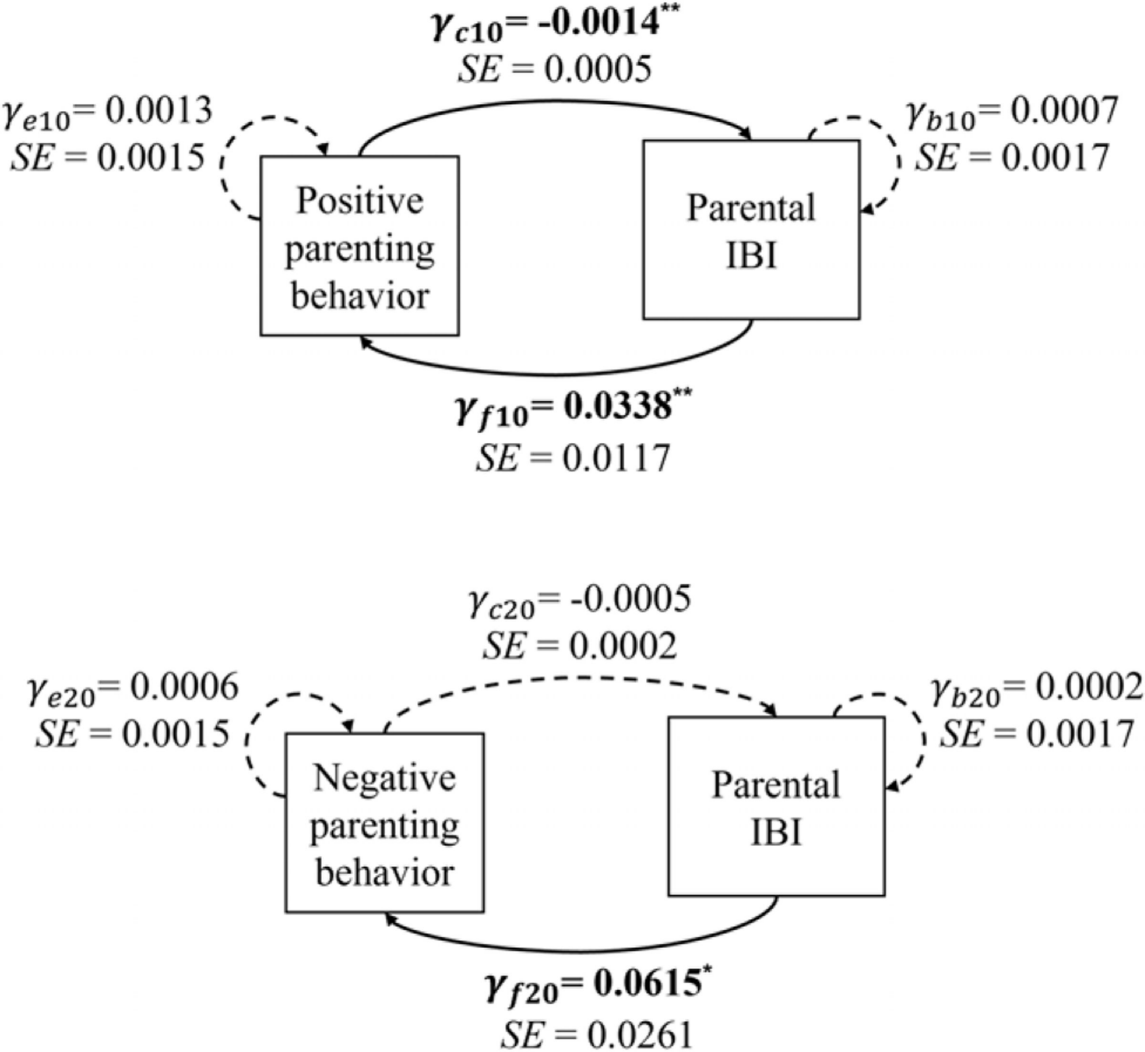
The dynamic associations between parents’ IBI and the local density of positive or negative parenting behaviors. *Note*. Each arrow indicates that how the momentary level of one variable (start of arrow) is associated with the momentary change of another variable (end of arrow). Solid lines represent statistically significant associations. * *p* < .05, ** *p* < .01, ** *p* < .001.

**Figure 3.**
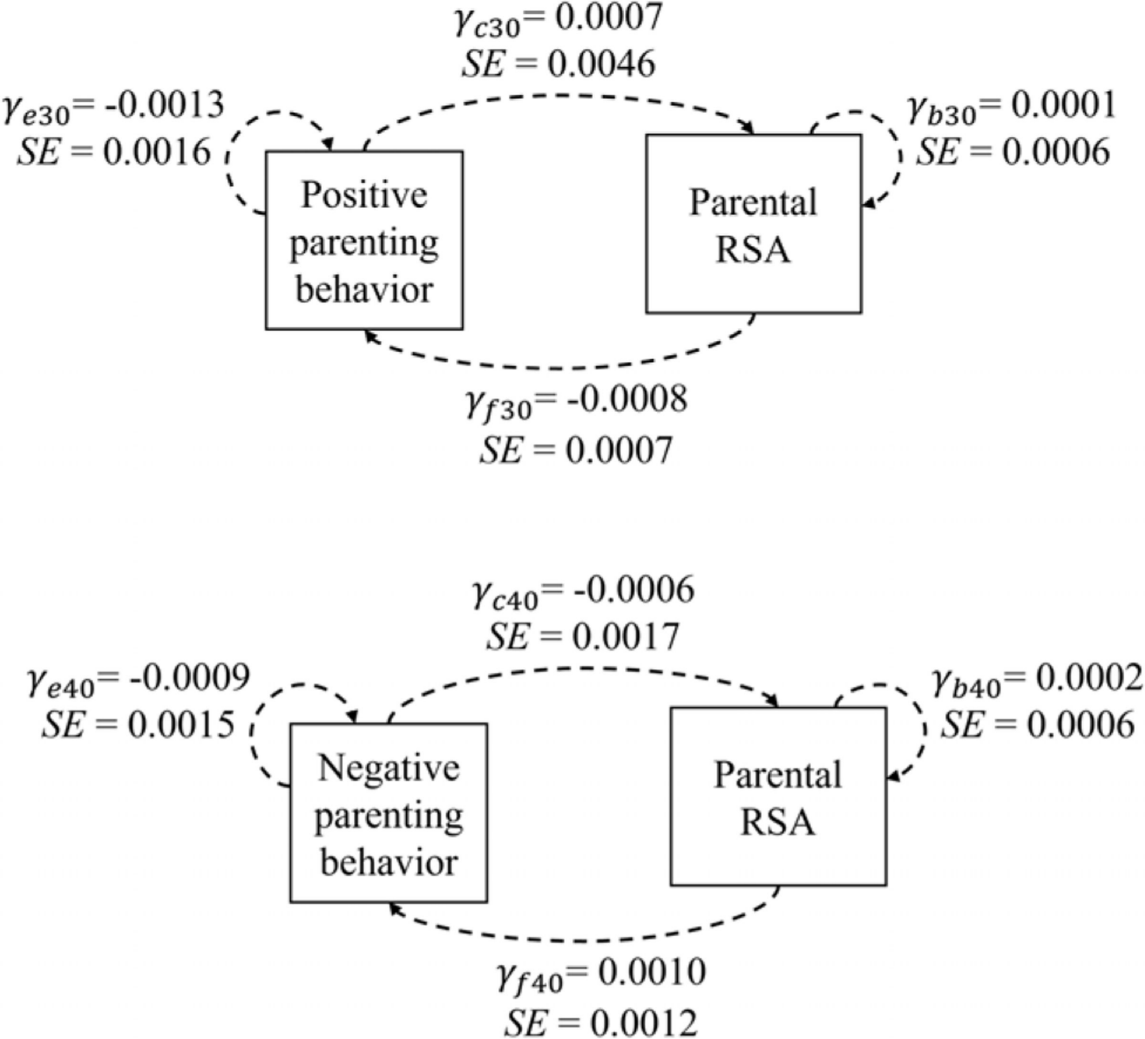
The dynamic associations between parents’ RSA and the local density of positive or negative parenting behaviors. *Note*. Each arrow indicates that how the momentary level of one variable (start of arrow) is associated with the momentary change of another variable (end of arrow). Solid lines represent statistically significant associations. * *p* < .05, ** *p* < .01, ** *p* < .001.

Results of models examining the sample-average dynamic associations between parent physiology and parenting behaviors were reported in the Supplementary Materials (Table S1 to S4) and illustrated in Figures 2 and 3. As shown in Figure 2, consistent with the hypothesized negative feedback loop, at moments when parents displayed more frequent positive behaviors, their IBI values tended to decrease ($\gamma_{c10}=-0.0014,SE=0.0005,p=.007$), reflecting momentary increases in cardiac arousal. In turn, greater cardiac arousal (i.e., shorter IBIs) relative to one’s task average predicted momentary decreases in positive parenting behaviors ($\gamma_{f10}=0.0338,SE=0.0117,p=.004$). Further, we found that greater cardiac arousal at a given moment in time was also associated with decreases in negative parenting behaviors ($\gamma_{f20}=0.0615,SE=0.0261,p=.02$), but negative parenting behaviors did not significantly predict momentary change in parents’ IBI ($\gamma_{c20}=-0.0005,SE=0.0002,p=.06$). To summarize, based on the current coding of parenting behaviors, results indicated that parents tended to experience increased cardiac arousal when they showed more positive verbalizations toward their children. Heightened arousal in turn predicted decreases in both positive and negative verbal engagement

In contrast to the significant IBI-parenting behavior dynamic associations observed, no significant sample-wide associations were found between parent RSA scores and parenting behavior. In other words, across the sample, momentary changes in parent RSA were not associated with the local density of positive ($\gamma_{c30}=0.0007,SE=0.0046,p=.88$) or negative ($\gamma_{c40}=-0.0006,SE=0.0017,p=.73$) parenting behaviors, nor did the momentary level of parent RSA predict changes in positive ($\gamma_{f30}=-0.0008,SE=0.0007,p=.25$)or negative parenting behaviors $\left(\gamma_{f40}=0.0010,SE=0.0012,p=.39\right)$ .

In addition to the dynamic associations between parent physiology and behavior, we did not find overall increasing or decreasing trends or patterns of intrinsic dynamics in IBI, RSA, or parenting behaviors, indicated by statistically nonsignificant intercepts (i.e., $\gamma_{a10}$ to $\gamma_{a40}$, and $\gamma_{d10}$ to $\gamma_{d40},ps>.30$ ) and parameters of how the variables’ momentary changes were predicted by their own concurrent levels (i.e., *γ*_*b*10_ to *γ*_*b*40_, and *γ*_*e*10_ to *γ*_*e*40_, *p*s > .30, see Tables S1 to S4).

### Inter-individual differences in parent physiology-behavior dynamics

#### Moderators of IBI and positive parenting dynamic associations

Several parent characteristics associated with maltreatment risks moderated the dynamic associations between IBI and positive parenting (see Table 2), specifically parents’ harsh child attributions, responses to positive emotion cues, and mental health symptoms. Among parents who held harsher child attributions, there were no significant dynamic associations between their IBI and positive parenting behaviors (at $M+SD:c_{1i}=-0.0003,p=.70$ and $f_{1i}=0.0085,p=.62$). In contrast, positive parenting behaviors predicted an increase in cardiac arousal and higher arousal predicted a decrease in positive behaviors only among parents with average (see estimates of $\gamma_{c10}$ and $\gamma_{c10}$) or *less* harsh attributions of their child (at $M–SD:c_{1i}=-0.0026,p=.001$ and $f_{1i}=0.0589,p=.004$). Regarding responses to emotion cues, parent accuracy in identifying positive emotions moderated how positive parenting behaviors predicted changes in arousal. The dynamic association linking more positive parenting behaviors with a momentary increase in cardiac arousal was only observed in parents who were less accurate at perceiving happy facial expressions (at $M–SD,c_{1i}=-0.0022,p=.002$), but not among parents with greater accuracy (at $M+SD,c_{1i}=0.0022,p=.82$). Neither parent false alarms to anger nor child-focused parenting stress moderated how positive parenting predicted changes in IBI.

Parent mental health symptoms moderated how parents’ IBI level predicted momentary changes in positive parenting behaviors. Parents with average (see the estimate of $\gamma_{f10}$) or lower levels of mental health concerns (at $M-SD:f_{1i}=0.0641,p<.001$) were more likely to disengage from positive verbalizations when experiencing heightened cardiac arousal (i.e., shorter IBIs). However, among parents with higher levels of mental health symptoms, no relation was observed between cardiac arousal and the momentary changes in positive parenting behaviors (at $M+SD:f_{1i}=0.0016,p=.92$). The moderating effect of mental health symptoms was similar for depressive and anxiety symptoms when examined separately. Although mental health symptoms and inhibitory control problems were correlated at *r* = .54 (*p* < .001), the latter was not a significant moderator of the dynamic associations between positive parenting and IBI.

#### Moderators of IBI and negative parenting dynamic associations

Next, we examined moderators of the associations between negative parenting behaviors and parents’ cardiac arousal. On average, $c_{2i}$did not differ significantly from 0, indicating that the local density of negative parenting behaviors did not predict dynamic changes in parents’ cardiac arousal. However, the value of $c_{2i}$ aried by the total frequency of negative parenting behaviors. Among parents who showed fewer overall instances of negative behaviors, their negative behaviors predicted momentary increases in cardiac arousal (i.e., decreases in IBI; at *M* – *SD* for task-average frequency of negative parenting behaviors,$c_{2i}=-0.0012,p=.002$).

Furthermore, several parent characteristics were significant moderators of how negative parenting behaviors changed at moments of heightened cardiac arousal (i.e., the value of *f*_2*i*_). First, parents’ speed of responding to happy facial cues moderated this dynamic association, and post hoc analyses suggested that the effect was not specific to the reaction time during the Happy-target block but was true for reaction times to correct hit trials during *all* emotion-target blocks. Among parents with average or longer reaction times (i.e., slower responding), greater cardiac arousal (lower IBI) predicted a momentary decrease in negative parenting behaviors, whereas for parents with shorter reaction times (e.g., *M* – *SD*), IBI levels did not predict changes in negative parenting behaviors. In other words, parents who took more time before correctly responding to emotional faces – whether positive or negative ones – were more likely to disengage from negative or controlling verbalizations when they experienced greater arousal.

Likewise, child-focused parenting stress moderated the association between arousal and momentary changes in negative parenting. Among parents with average or low levels of child-focused parenting stress (see $\gamma_{f20}$ for an estimate of *f*_2*i*_ when parenting stress is at sample mean; at *M* – *SD* for parenting stress,$f_{2i}=0.1355,p<.001$), reductions in negative parenting occurred at moments of greater cardiac arousal (shorter IBI). In contrast, IBI levels were unrelated to any change in negative behaviors among parents who reported high child-focused parenting stress (at $M–SD,f_{2i}=-0.0031,p=.93$). Mental health symptoms and inhibitory control problems also showed similar moderation effects. At lower levels of mental health symptoms or inhibitory control problems, greater arousal (i.e., shorter IBI) predicted decreases in negative parenting behaviors, whereas for parents who reported greater mental health symptoms and/or inhibitory control problems, IBI levels were unrelated to any change in negative parenting.

#### Moderators of RSA and positive/negative parenting dynamic associations

As shown in Table 3, although no sample-wide RSA-behavior dynamic associations were observed, some significant moderation effects emerged for the association between RSA and positive parenting. Among parents who showed more total positive parenting behaviors, their positive parenting tended to increase at moments of lower RSA (at *M + SD* for total positive parenting behaviors frequency,$f_{3i}=-0.0030,p<.001$). Similarly, parents with better inhibitory control also showed a pattern of increasing positive parenting behaviors when their RSA was lower (indicating parasympathetic withdrawal and thus greater arousal; at *M* – *SD* for inhibitory control problems,$f_{3i}=-0.0021,p=.02$). RSA levels were unrelated to momentary changes in positive parenting among those showing fewer positive behaviors in total ($M–SD,f_{3i}=0.0015,p=.10$) or reporting greater inhibitory control problems ($M+SD,f_{3i}=0.0003,p=.69$).

Furthermore, parent responses to emotion cues moderated the bi-directional associations between RSA and positive parenting dynamics. Among parents who were more accurate in identifying happy facial emotion (hit rate above sample median), positive parenting tended to increase at moments of lower RSA $\left(f_{3i}=-0.0028,p=.008\right)$. Additionally, these parents also showed a trend of increases in RSA when they were engaging in positive parenting behaviors, with the simple slope coefficient approaching statistical significance (when hit rate was above sample median, $c_{3i}=0.0148,p=.07$). For parents with lower accuracy in identifying positive emotion cues, no significant dynamic RSA-positive parenting associations were evident (at hit rate below sample median,$f_{3i}=0.0003,p=.74,c_{3i}=-0.0066,p=.28$).

In summary, a dynamic pattern of increasing positive parenting behaviors when experiencing parasympathetic withdrawal was observed among parents who displayed more positive parenting behaviors across the child-led play task, better inhibitory control ability, and greater accuracy in recognizing and responding to positive emotion cues. Parents with greater accuracy in reacting to positive emotion cues were also more likely to show an increase in RSA as they engaged in positive behaviors. No other parent characteristics examined in this study moderated the dynamic associations between RSA and positive parenting. Moreover, no significant moderating effects were found for the dynamic associations between RSA and negative parenting (see Table 3).

## Discussion

Building on previous work conceptualizing parents’ self-regulation as dynamic processes that continuously modulate their internal states and parenting behaviors, the present study tested hypotheses around how dynamic associations between physiology and behavior may reflect regulatory processes and difficulties in child welfare-involved parents. Based on data collected during a play task where parents should follow their children’s lead, results of ordinary differential equations revealed dynamic associations between parents’ cardiac arousal and parenting behaviors on a moment-to-moment basis, consistent with the hypothesized conflict between their internal states and parental role. The findings suggest that carrying out positive parenting efforts may be physiologically “taxing” for many parents in this sample, and parents tended to disengage from verbal interactions at moments of heightened cardiac arousal. Meanwhile, parents demonstrating more positive parenting behaviors in total showed regulatory patterns in which momentary parasympathetically-mediated arousal predicted an increase in positive verbal engagement with the child. This study took an ecologically-valid approach to understanding how parents’ physiological responses may facilitate or interfere with their ability to support children on a moment-to-moment basis, thus identifying mechanisms that should be targeted in interventions. It also expands the conceptual and methodological framework for assessing parents’ self-regulatory processes in research and clinical settings.

### Physiology-behavior dynamic associations in parenting

Consistent with our hypothesis, the dynamic associations between parents’ overall cardiac arousal and positive parenting behaviors manifested as a negative feedback loop in this child welfare-involved sample. When parents engaged in more positive verbalizations with their child, their cardiac arousal tended to increase. Meanwhile, heightened cardiac arousal predicted momentary decreases in positive verbal engagement. Similar dynamic associations were previously reported in a sample of physically abusive parents, though observed within parent RSA-behavior dynamic associations across 30-s segments of dyadic interaction (Skowron et al., 2013), not via moment-to-moment IBI-behavior linkages observed here. Interestingly, greater cardiac arousal also predicted decreases in negative parenting behaviors in our sample, suggesting that parents might be withdrawing from verbal interactions in general in the midst of arousal, rather than increasing harsh control as we originally hypothesized and as observed by Skowron et al. (2013) among physically abusive parents. It is worth noting that a high proportion of parents in the current sample (41.7%) entered the study with a prior record of child neglect, per the substantiated records of maltreatment. Although the specific reasons why the rest of the sample was involved with child welfare were unknown to us, parents who have been referred to child welfare services for documented child neglect may tend to withdraw from their children when experiencing heightened physiological arousal. This is also consistent with the perspective derived from Bowen’s theory (1978), which suggests that parents may show emotional and behavioral detachment to avoid distress when they have difficulty regulating their own arousal (Smith, 2003), increasing the risk for neglect. Alternately, it is also possible that these parents restrained themselves from showing negative verbalizations in front of the camera.

Regarding RSA-behavior dynamic associations, no sample-wide statistically significant patterns were observed. However, moderator analyses revealed that among parents who demonstrated more positive parenting behaviors overall, lower RSA predicted momentary increases in positive parenting behaviors. This pattern was also observed in a previous study among low-risk mothers (Zhang et al., 2022), suggesting that when parents’ physiological equilibrium has been perturbed during parenting, evidenced by parasympathetic withdrawal (i.e., lower RSA relative to one’s task average), these parents may increase positive parenting behaviors as an effort to cope with the situation. Furthermore, our post hoc analysis found that parents who self-reported more affirming parenting in daily life also demonstrated this pattern of increasing positive verbalizations at moments of lower RSA (see Table S5 in Supplementary Materials). In fact, these parents showed bi-directional dynamic associations between RSA and positive behaviors, such that when engaging in positive parenting behaviors, their RSA tended to increase (indicating physiological calm). These findings again were consistent with a feedback loop theorized to reflect parents’ self-regulatory functioning (i.e., engaging in child-centered behaviors to help parents themselves regulate physiological reactivity) and evident in the abovementioned low-risk sample.

Physiology and behavior are influenced by many factors, and no causal inferences should be made based on the current findings. Nonetheless, these temporal relations between parental physiology and parenting behaviors may capture patterns reflecting regulatory processes or difficulties that point to potentially different roles of overall cardiac reactivity versus parasympathetic inputs in parenting-related dynamics. Momentary cardiac arousal, reflected in lower IBI values, was involved in dynamic patterns consistent with difficulties in sustaining positive parenting behaviors observed in this at-risk sample. On the contrary, momentary parasympathetic innervation of cardiac arousal, reflected in lower RSA, was involved in dynamic patterns consistent with engaging positive parenting behaviors to help regulate arousal, and such patterns were only evident in a subgroup of our sample that demonstrated indicators of competence with positive parenting (i.e., showing more positive behaviors during the task and reporting more affirming parenting in daily life). It is possible that sympathetic inputs were driving the results with IBI, capturing how fight-or-flight responses were activated by parenting demands and interfering with positive parenting behaviors. In the meantime, consistent with the polyvagal theory (Porges, 2007) and a dynamic systems view of parent physiology-behavior regulation (Zhang et al., 2022), flexible withdrawal and restoration of parasympathetic control may be involved in actively coping with parenting challenges and balancing internal and external demands. However, we did not collect any sympathetic measure that could support similar dynamic analyses, and future research is needed to further investigate the role of sympathetic reactivity and its interplay with parasympathetic inputs in the regulation of parenting.

Notably, we observed the IBI- and RSA-behavior dynamic associations during a child-led play task that did not impose external challenges on the dyads. Here, positive and negative parenting behaviors were operationalized based on the task context that demanded parents’ efforts with reciprocity and support (e.g., giving commands may be appropriate and effective in discipline situations, but was viewed as controlling and thus inappropriate when parents were instructed to support their children’s interests in this task). However, as contextual demands may influence the implication of physiological responses and how self-regulation processes unfold, it is important that future research explores whether these patterns replicate or vary systematically in other parenting contexts, especially ones with more significant stressors and/or more structured behavioral demands (e.g., disciplinary situations, joint problem-solving). Compared to the current task where parents were only given a general set of instructions (i.e., to follow their child’s lead in the play), other compliance-based tasks present more clear and pressing demands (e.g., getting the child to do or not to do something) and may require autonomic responses to both facilitate active engagement and prevent the dominance of fight-or-flight responses (e.g., sympathetic–parasympathetic coactivation; Miller et al., 2015). Such context-specific demands on dynamic physiological changes should be investigated in future research. Meanwhile, we also recognize that beyond the general task context, each parent faces unique, time-varying demands that depend on how the interaction unfolds and how they perceive their children and the relationship (e.g., at-risk parents may perceive free play with their child as highly demanding). By accounting for real-time parenting, the current analyses aimed to capture such “micro contexts” in association with demands placed on parents’ physiological system. This might also explain why the findings based on this free-play task partially replicated findings from previous studies using more challenging tasks.

### Inter-individual differences in the dynamic associations and parent characteristics

While none of the parent characteristics was correlated with parents’ task-average physiology and behavior frequency, several were related to inter-individual differences in the dynamic physiology-behavior associations. The IBI-behavior negative feedback loop linking cardiac arousal with dampened positive verbalizations was more evident among parents with gentler attributions about their children and lower mental health symptoms. At first, this was puzzling to us because harsh attributions and mental health symptoms are well-recognized risk factors for child maltreatment (Azar & Weinzierl, 2005; Milner, 2003; Stith et al., 2009), and thus we reasoned that they would predict greater regulatory difficulties that interfere with appropriate parenting. However, it is important to recognize that all families in this sample were already involved with child welfare and thus showed some form of dysfunction. It is possible that the regulatory difficulties reflected in the negative feedback loop explained why some parents, despite not having those well-recognized risk factors (i.e., harsh child attributions and mental health symptoms), ended up in this high-risk group. Those parents might have particular difficulty in showing positivity and support toward their children’s interests and needs. This speculation was supported by the findings that (a) parents who showed less accuracy in identifying positive emotion cues (i.e., happy facial expressions) were more likely to experience increased arousal when engaging in positive parenting, as well as by (b) post hoc analyses showing that parents who self-reported less affirming parenting in daily life demonstrated greater dynamic regulatory difficulties reflected in the associations between IBI and positive behaviors (see Table S5 in Supplementary Materials for details of the measure and the post hoc results). In comparison, several favorable parent characteristics (i.e., more observed and self-reported positive parenting behaviors, better self-reported inhibitory control, and greater accuracy in identifying positive emotion cues) moderated RSA-positive parenting dynamic associations. Specifically, they were related to dynamic patterns of increasing positive parenting behaviors at moments of parasympathetic withdrawal.

Regarding negative parenting, several parent characteristics, including lower levels of child-focused parenting stress, mental health symptoms, and inhibitory control difficulties, as well as slower reactions to emotional faces (potentially reflecting less impulsive responses to emotion cues), were related to a greater likelihood to disengage from negative verbalizations when parents experience greater cardiac arousal. In contrast, at higher levels of those risk factors or faster reaction times, parents’ cardiac arousal and negative parenting behaviors were not dynamically associated. Although the presence of the camera may have played a role, it may be adaptive for parents to temporarily disengage from the interaction at moments of higher arousal. For example, a technique in mindful parenting involves pausing before reacting when feeling upset (Duncan et al., 2009), which can prevent parents from acting upon impulses of harsh or controlling behaviors. The findings suggest that this may be harder for parents who have higher levels of child-focused parenting stress, mental health symptoms, or difficulty inhibiting impulses. None of the parent characteristics moderated the associations between RSA and negative parenting, indicating no evidence of any subgroup in which RSA may be involved in sustaining or provoking negative parenting behaviors.

It is worth noting that parents’ mental health symptoms moderated how *both* positive and negative parenting behaviors changed as a function of their momentary cardiac arousal, and such effects did not differ for depression versus anxiety. Thus, in addition to the interpretations we offered above, it is also possible that for parents who experience more pervasive psychological distress, their behaviors toward children in general (regardless of positive or negative) were less dependent on their momentary physiological states. Previous research has found that maternal depression was associated with more negative emotions oriented toward parents’ own demands as well as less supportive behaviors toward their children (Dix et al., 2014). However, it is unclear whether and how those characteristics may manifest in parents’ moment-to-moment regulatory processes, so more research is still needed to understand this phenomenon.

### Limitations, future directions, and implications

The interpretation and generalization of the findings should take into consideration the specific context of the analyses (e.g., child welfare-involved parents of 3- to 7-year-olds) as well as some limitations. First, the observational coding of parenting behaviors focused on specific types of positive and negative utterances. Although the DPICS is a well-validated system (Eyberg & Funderburk, 2011), it may not capture the full range of behaviors. For example, when parents disengaged verbally at moments of heightened cardiac arousal, they might have engaged in nonverbal expressions of attentiveness and affection (e.g., smiling, hugging) or negativity (e.g., stonewalling). Additionally, the event-based coding allowed us to quantify the local density of behaviors but may not fully capture the quality of those behaviors, such as the degree of positive affection embedded in parents’ verbal expressions or how responsive parents were to children’s momentary interests and needs. Future studies could extend the current approach by incorporating dimensions of parenting quality into the analyses (e.g., parents may be able to keep using supportive verbalizations but with a decrease in affective quality when they experience heightened arousal). Second, observational data on children’s behaviors, especially their engagement during the play and whether they demonstrated behaviors that would be challenging for parents to handle, was not available for the current analyses. Those behaviors may be an important factor, and potentially a source of variance, in parents’ emotions and self-regulation processes in the context of parenting. Furthermore, including children in the models can help researchers understand how parents’ self-regulation may support their ability to scaffold their children’s self-regulation. For example, previous studies have observationally coded dynamic changes in children’s challenging behaviors and negative affect, showing that they may predict parents’ momentary emotional reactivity and be regulated by parents’ behaviors (e.g., Lorber & Slep, 2005; Zhang et al., 2022). Another body of research has illustrated dynamic concordance between parents’ and children’s physiological responses during interactions, although more work is needed to understand its correspondence with behavioral processes and how parents contribute to a co-regulation space that facilitates growth in dyadic functioning and children’s regulatory skills. Fourth, we did not collect any measure of sympathetic activity that could support similar dynamic analysis (e.g., skin conductance level). In this study, no significant sample-wide physiology-behavior dynamic associations were found for RSA, an indicator of parasympathetic activity. Patterns emerged with IBI that reflected overall ANS arousal shaped by both sympathetic and parasympathetic inputs, which may limit its physiological interpretability. Due to a lack of available measures, we could not directly test the role of sympathetic activity in parents’ regulatory difficulties, which warrants examination in future studies. Finally, as mentioned earlier, we do not know the specific reasons why parents in this sample were involved with the child welfare system. Although the measures have provided a picture of their cognitive and emotional characteristics, it would be helpful to understand their specific parenting concerns and examine how they may be related to real-time regulatory difficulties.

Despite the limitations, this study illustrates an innovative approach to conceptualizing and assessing parents’ regulatory difficulties that may contribute to parenting risks. We identified bi-directional, dynamic associations between parents’ moment-to-moment physiological arousal and parenting behaviors, which reflect a conflict between parents’ internal arousal and parenting demands, revealing why it may be hard for some parents to sustain positive verbal engagement with their children even in typically non-challenging parenting contexts. These results provided a foundation for future work to identify components or features of parenting behaviors (e.g., emotional engagement) that are more closely associated with demands on the physiological system, and specific neurophysiological mechanisms underlying such associations. The findings further linked parent characteristics to the dynamic associations between parents’ cardiac arousal and parenting behaviors (but not to task-average physiology or behavior frequency). This highlights the unique value of a dynamic approach for understanding mechanisms that may connect commonly-known risk factors to how at-risk parenting unfolds in real-time during parent-child interaction.

## Supplementary Material

1

## Figures and Tables

**Table 1.** Descriptive statistics of study variables reflecting inter-individual differences

	1	2	3	4	5	6	7	8	9	10	11	12	13
1. Child age (years)	-												
2. Parent task-average IBI	.01	-											
3. Parent task-average RSA	−.09	.65***	-										
4. Positive behavior frequency	−.29***	.07	.04	-									
5. Negative behavior frequency	−.10	.13	.13	.15*	-								
6. Harsh child attributions	.00	.00	−.07	.07	−.03	-							
7. Child-focused parenting stress	.02	−.02	.04	−.10	.10	.06	-						
8. Hit rate for positive emotion	−.11	−.06	−.04	−.03	.09	.07	.06	-					
9. Reaction time (Happy-target, Neutral-distractor)	−.07	.01	−.12	−.08	−.13	.07	−.06	−.29***	-				
10. False alarm rate to anger	−.12	−.04	.03	.12	.00	−.03	.07	.00	−.16*	-			
11. Reaction time (Neutral-target, Angry-distractor)	−.02	.10	.02	−.10	−.06	.05	−.05	−.17*	.39***	−.18*	-		
12. Mental health symptoms	.12	−.07	−.09	−.06	−.07	.03	.29***	.02	.06	.04	.11	-	
13. Inhibitory control problems	.10	−.05	−.12	.00	−.02	−.02	.26***	.01	−.07	.09	−.06	.54***	-
*N*	204	202	202	203	203	202	203	198	198	199	196	204	204
*M*	4.76	0.76	5.36	5.27	23.16	71.19	64.65	0.35	0.41	0.40	0.43	55.83	55.60
*SD*	1.40	0.12	1.39	4.44	11.55	45.66	24.75	0.48	0.03	0.49	0.03	9.24	10.05
*Min*	3.00	0.54	2.10	0	0	0	3	0	0.32	0	0.33	40	37
*Max*	8.00	1.24	8.93	27	67	200	99	1	0.49	1	0.51	80	82

*Note*. Task-average inter-beat interval (IBI) and respiratory sinus arrhythmia (RSA) levels were calculated by averaging the second-by-second estimates. Positive/negative behavior frequency represents frequency of positive/negative parental behaviors across the entire task. Hit rate for positive emotion (whether parents correctly pressed “go” in response to happy faces for more than 70% of the “happy = go” trials) and false alarm rate to anger (whether parents mistakenly pressed “go” in response to neutral faces for any “anger = go” trial) are dichotomized variables. Reaction times represent the average reaction times (in seconds) to correct hit trials during the two blocks of interest.

* *p* < .05,

** *p* < .01,

*** *p* < .001.

**Table 2.** Parental characteristics moderating the dynamic associations between parental IBI and parenting behaviors

Predictor	Parameters representing intra-individual dynamic associations							
	*c*_1*i*_ (Positive Behavior→IBI)		*f*_1*i*_ (IBI→Positive Behavior)		*c*_2*i*_ (Negative Behavior→IBI)		*f*_2*i*_ (IBI→ Negative Behavior)	
	Coefficient (*SE*)	*p*	Coefficient (*SE*)	*p*	Coefficient (*SE*)	*p*	Coefficient (*SE*)	*p*
Positive behavior frequency	−0.0003 (0.0005)	.60	0.0035 (0.0119)	.77	-	-	-	-
Negative behavior frequency	-	-	-	-	0.0006* (0.0002)	.01	−0.0481 (0.0253)	.06
Harsh child attributions	0.0011* (0.0005)	.04	−0.0252* (0.0123)	.04	0.0001 (0.0002)	.57	−0.0295 (0.0268)	.27
Child-focused parenting stress	−0.0003 (0.0005)	.54	−0.0018 (0.0121)	.88	0.0004 (0.0002)	.08	−0.0693** (0.0262)	.008
Hit rate for positive emotion	0.0025* (0.0012)	.04	−0.0425 (0.0251)	.09	−0.0001 (0.0005)	.92	0.0199 (0.0570)	.73
Reaction time (Happy-target, Neutral-distractor)	0.0010 (0.0006)	.10	−0.0173 (0.0119)	.14	−0.0004 (0.0003)	.10	0.0820** (0.0270)	.002
False alarm rate to anger	−0.0012 (0.0011)	.31	−0.0117 (0.0240)	.63	−0.0004 (0.0005)	.41	0.0279 (0.0554)	.61
Reaction time (Neutral-target, Angry-distractor)	0.0004 (0.0006)	.43	−0.0231 (0.0121)	.06	−7.42E-6 (0.0002)	.98	0.0190 (0.0278)	.49
Mental health symptoms	0.0007 (0.0005)	.17	−0.0312* (0.0121)	.01	0.0004 (0.0002)	.14	−0.0709** (0.0265)	.008
Inhibitory control problems	−9.92E-6 (0.0005)	.99	−0.0052 (0.0122)	.67	0.0003 (0.0002)	.22	−0.0549* (0.0263)	.04

*Note*. All predictors were standardized using sample means and standard deviations. Positive or negative behavior frequency refers to the average frequency of corresponding parenting behaviors across the task.

* *p* < .05,

** *p* < .01,

*** *p* < .001.

**Table 3.** Parental characteristics moderating the dynamic associations between parental RSA and parenting behaviors

Predictor	Parameters representing intra-individual dynamic associations							
	*c*_3*i*_ (Positive Behavior→RSA)		*f*_3*i*_ (RSA→Positive Behavior)		*c*_4*i*_ (Negative Behavior→RSA)		*f*_4*i*_ (RSA→ Negative Behavior)	
	Coefficient (*SE*)	*p*	Coefficient (*SE*)	*p*	Coefficient (*SE*)	*p*	Coefficient (*SE*)	*p*
Positive behavior frequency	0.0035 (0.0045)	.44	−0.0022*** (0.0006)	< .001	-	-	-	-
Negative behavior frequency	-	-	-	-	−0.0012 (0.0017)	.47	0.0016 (0.0012)	.17
Harsh child attributions	−0.0024 (0.0048)	.61	0.0005 (0.0006)	.41	0.0001 (0.0017)	.94	−0.0009 (0.0012)	.49
Child-focused parenting stress	−0.0051 (0.0045)	.25	0.0006 (0.0006)	.37	−0.0002 (0.0017)	.89	−0.0007 (0.0013)	.59
Hit rate for positive emotion	0.0214* (0.0102)	.04	−0.0031* (0.0013)	.02	−0.0061 (0.0038)	.11	0.0034 (0.0026)	.20
Reaction time (Happy-target, Neutral-distractor)	−0.0027 (0.0051)	.60	0.0007 (0.0006)	.28	−0.0006 (0.0018)	.74	−0.0003 (0.0012)	.83
False alarm rate to anger	0.0011 (0.0100)	.92	0.0004 (0.0014)	.76	0.0019 (0.0037)	.60	−0.0004 (0.0026)	.88
Reaction time (Neutral-target, Angry-distractor)	0.0044 (0.0051)	.39	−0.0002 (0.0007)	.81	−0.0004 (0.0018)	.80	0.0004 (0.0013)	.73
Mental health symptoms	−0.0061 (0.0047)	.19	0.0012 (0.0006)	.07	−0.0002 (0.0017)	.91	0.0004 (0.0013)	.73
Inhibitory control problems	−0.0075 (0.0046)	.10	0.0012* (0.0006)	.04	0.0004 (0.0017)	.81	−0.0003 (0.0012)	.81

*Note*. All predictors were standardized using sample means and standard deviations. Positive or negative behavior frequency refers to the average frequency of corresponding parenting behaviors across the task.

* *p* < .05,

** *p* < .01,

*** *p* < .001.

## References

[R1] Abidin RR. (2012). Parenting stress index (4th edn). PAR.

[R2] Azar ST, & Weinzierl KM. (2005). Child maltreatment and childhood injury research: A cognitive behavioral approach. Journal of Pediatric Psychology, 30(7), 598–614. 10.1093/jpepsy/jsi046 16166248

[R3] Barnett D, Manly JT, & Cicchetti D. (1993). Defining child maltreatment: The interface between policy and research. In Cicchetti D, & Toth SL (Eds.), Child abuse, child development, and social policy (pp. 7–73). Ablex.

[R4] Benjamin LS, Rothweiler JC, & Critchfield KL. (2006). The use of Structural Analysis of Social Behavior (SASB) as an assessment tool. Annual Review of Clinical Psychology, 2(1), 83–109. 10.1146/annurev.clinpsy.2.022305.095337 17716065

[R5] Berntson GG, Cacioppo JT, & Quigley KS. (1993). Respiratory sinus arrhythmia: Autonomic origins, physiological mechanisms, and psycho-physiological implications. Psychophysiology, 30(2), 183–196. 10.1111/J.1469-8986.1993.TB01731.X 8434081

[R6] Berntson GG, Quigley KS, & Lozano D. (2007). Cardiovascular psychophysiology. In Cacioppo JT, Tassinary LG, & Berntson GG (Eds.), Handbook of psychophysiology (3rd ed. pp. 182–210). Cambridge University Press.

[R7] Borelli JL, Burkhart ML, Rasmussen HF, Smiley PA,& Hellemann G. (2018). Children’s and mothers’ cardiovascular reactivity to a standardized laboratory stressor: Unique relations with maternal anxiety and overcontrol. Emotion, 18(3), 369–385. 10.1037/emo0000320 28481573

[R8] Butler EA, Wilhelm FH, & Gross JJ. (2006). Respiratory sinus arrhythmia, emotion, and emotion regulation during social interaction. Psychophysiology, 43(6), 612–622. 10.1111/j.1469-8986.2006.00467.x 17076818

[R9] Camilo C, Vaz Garrido M, & Calheiros MM. (2021). Recognizing children’s emotions in child abuse and neglect. Aggressive Behavior, 47(2), 161–172. 10.1002/ab.21935 33164223

[R10] Chow SM. (2019). Practical tools and guidelines for exploring and fitting linear and nonlinear dynamical systems models. Multivariate Behavioral Research, 54(5), 690–718. 10.1080/00273171.2019.1566050 30950646 PMC6736768

[R11] Cole PM, Bendezú JJ, Ram N, & Chow S-M. (2017). Dynamical systems modeling of early childhood self-regulation. Emotion, 17(4), 684–699. 10.1037/emo0000268 28080091 PMC5882214

[R12] de Haan-Rietdijk S, Voelkle MC, Keijsers L, & Hamaker EL. (2017). Discrete- vs. continuous-time modeling of unequally spaced experience sampling method data. Frontiers in Psychology, 8, 1849. 10.3389/fpsyg.2017.01849 29104554 PMC5655034

[R13] Derogatis LR. (2001). Brief Symptom Inventory (BSI)-18: Administration, scoring and procedures manual. NCS Pearson.

[R14] Dix T. (1991). The affective organization of parenting: Adaptive and maladaptative processes. Psychological Bulletin, 110(1), 3–25. 10.1037/0033-2909.110.1.3 1891517

[R15] Dix T, Moed A, & Anderson ER. (2014). Mothers’ depressive symptoms predict both increased and reduced negative reactivity: Aversion sensitivity and the regulation of emotion. Psychological Science, 25(7), 1353–1361. 10.1177/0956797614531025 24796661

[R16] Dubowitz H, Kim J, Black MM, Weisbart C, Semiatin J, & Magder LS. (2011). Identifying children at high risk for a child maltreatment report. Child Abuse & Neglect, 35(2), 96–104. 10.1016/j.chiabu.2010.09.003 21376396

[R17] Duncan LG, Coatsworth JD, & Greenberg MT. (2009). A model of mindful parenting: Implications for parent-child relationships and prevention research. Clinical Child And Family Psychology Review, 12(3), 255–270. 10.1007/s10567-009-0046-3/figures/1 19412664 PMC2730447

[R18] Eyberg SM, & Funderburk B. (2011). Parent-child interaction therapy protocol. PCIT International.

[R19] Eyberg SM, Nelson MM, Ginn NC, Bhuiyan N, & Boggs SR. (2013). Dyadic parent-child interaction coding system (DPICS): Comprehensive manual for research and training (4th edn). PCIT International.

[R20] Fanti KA, & Henrich CC. (2010). Trajectories of pure and co-occurring internalizing and externalizing problems from age 2 to age 12: Findings from the National institute of Child Health and Human Development Study of Early Child Care. Developmental Psychology, 46(5), 1159–1175. 10.1037/a0020659 20822230

[R21] Feldman R, Magori-Cohen R, Galili G, Singer M, & Louzoun Y. (2011). Mother and infant coordinate heart rhythms through episodes of interaction synchrony. Infant Behavior and Development, 34(4), 569–577. 10.1016/j.infbeh.2011.06.008 21767879

[R22] Ferrey AE, Santascoy N, McCrory EJ, Thompson-Booth C, Mayes LC, & Rutherford HJV. (2016). Motivated attention and reward in parenting. Parenting: Science and Practice, 16(4), 284–301. 10.1080/15295192.2016.1184928

[R23] Francis KJ, & Wolfe DA. (2008). Cognitive and emotional differences between abusive and non-abusive fathers. Child Abuse & Neglect, 32(12), 1127–1137. 10.1016/j.chiabu.2008.05.007 19036447

[R24] Frodi AM, & Lamb ME. (1980). Child abusers’ responses to infant smiles and cries. Child Development, 51(1), 238–238. 10.2307/1129612 7363736

[R25] Gatzke-Kopp L, & Ram N. (2018). Developmental dynamics of autonomic function in childhood. Psychophysiology, 55(11), e13218. 10.1111/psyp.13218 30059155

[R26] Gatzke-Kopp L, Zhang X, Creavey KL, & Skowron EA. (2022). An event-based analysis of maternal physiological reactivity following aversive child behaviors. Psychophysiology, 59(11), e14093. 10.1111/PSYP.14093 PMC9532353 35567524

[R27] Green LM, Genaro BG, Ratcliff KA, Cole PM, & Ram N. (2023). Investigating the developmental timing of self-regulation in early childhood. International Journal of Behavioral Development, 47(2), 101–110. 10.1177/01650254221111788 36865026 PMC9974174

[R28] Hildyard K, & Wolfe D. (2007). Cognitive processes associated with child neglect. Child Abuse & Neglect, 31(8), 895–907. 10.1016/j.chiabu.2007.02.007 17804068

[R29] Kreibig SD. (2010). Autonomic nervous system activity in emotion: A review. Biological Psychology, 84(3), 394–421. 10.1016/j.biopsycho.2010.03.010 20371374

[R30] Lavi I, Ozer EJ, Katz LF, & Gross JJ. (2021). The role of parental emotion reactivity and regulation in child maltreatment and maltreatment risk: A meta-analytic review. Clinical Psychology Review, 90, 102099. 10.1016/j.cpr.2021.102099 34752992

[R31] Levenson RW. (2014). The autonomic nervous system and emotion. Emotion Review, 6(2), 100–112. 10.1177/1754073913512003

[R32] Lorber MF, & Slep AMS. (2005). Mothers’ emotion dynamics and their relations with harsh and lax discipline: Microsocial time series analyses. Journal of Clinical Child and Adolescent Psychology, 34(3), 559–568. 10.1207/s15374424jccp3403_11 16026219 PMC1415277

[R33] Lunkenheimer E, Ram N, Skowron EA, & Yin P. (2017). Harsh parenting, child behavior problems, and the dynamic coupling of parents’ and children’s positive behaviors. Journal of Family Psychology, 31(6), 689–698. 10.1037/fam0000310 28333490 PMC5608615

[R34] Martínez CAG, Quintana AO, Vila XA, Touriño MJL, Rodríguez-Liñares L, Presedo JMR, & Penín AJM. (2017). Heart rate variability analysis with the R package RHRV. Springer. 10.1007/978-3-319-65355-6

[R35] McCanne TR, & Hagstrom AH. (1996). Physiological hyperreactivity to stressors in physical child abusers and individuals at risk for being physically abusive. Aggression and Violent Behavior, 1(4), 345–358. 10.1016/S1359-1789(96)00004-3

[R36] McKillop HN, & Connell AM. (2018). Physiological linkage and affective dynamics in dyadic interactions between adolescents and their mothers. Developmental Psychobiology, 60(5), 582–594. 10.1002/dev.21630 29748953

[R37] Miller JG, Chocol C, Nuselovici JN, Utendale WT, Simard M, & Hastings PD. (2013). Children’s dynamic RSA change during anger and its relations with parenting, temperament, and control of aggression. Biological Psychology, 92(2), 417–425. 10.1016/j.biopsycho.2012.12.005 23274169 PMC4055035

[R38] Miller JG, Kahle S, Lopez M, & Hastings PD. (2015). Compassionate love buffers stress-reactive mothers from fight-or-flight parenting. Developmental Psychology, 51(1), 36–43. 10.1037/a0038236 25329554 PMC4351818

[R39] Milner JS. (2003). Social information processing in high-risk and physically abusive parents. Child Abuse & Neglect, 27(1), 7–20. 10.1016/s0145-2134(02)00506-9 12510028

[R40] Nelson MM, & Olsen B. (2018). Dyadic Parent-Child Interaction Coding System (DPICS): An adaptable measure of parent and child behavior during dyadic interactions. In Niec L (Ed.), Handbook of parent-child interaction therapy (pp. 285–302). Springer.

[R41] Oliveira-Silva P, & Gonçalves ÓF. (2011). Responding empathically: A question of heart, not a question of skin. Applied Psychophysiology Biofeedback, 36(3), 201–207. 10.1007/s10484-011-9161-2 21717221

[R42] Ou L, Hunter MD, & Chow SM. (2019). What’s for dynr: A package for linear and nonlinear dynamic modeling in R. R Journal, 11(1), 91. 10.32614/rj-2019-012 34306735 PMC8297742

[R43] Pinheiro J, Bates D, DebRoy S, Sakar D, & R Core Team, nlme: Linear and nonlinear mixed effects models (R package ver. 3.1–149), 2017. https://cran.r-project.org/src/contrib/Archive/nlme/nlme_3.1-149.tar.gz

[R44] Porges SW. (2007). The polyvagal perspective. Biological Psychology, 74(2), 116–143. 10.1016/j.biopsycho.2006.06.009 17049418 PMC1868418

[R45] Ramsay JO, Hooker G, & Graves S. (2009). Functional data analysis with R and MATLAB. Springer.

[R46] Ramsay JO, & Silverman BW. (2005). Functional data analysis (2nd edn). Springer-Verlag.

[R47] Rath JF, & Fox LM. (2017). Brief symptom inventory. In Kreutzer JS, DeLuca J, & Caplan B (Eds.), Encyclopedia of clinical neuropsychology. Springer. 10.1007/978-0-387-79948-3_1977

[R48] Ravindran N, Zhang X, Green LM, Gatzke-Kopp LM, Cole PM, & Ram N. (2021). Concordance of mother-child respiratory sinus arrythmia is continually moderated by dynamic changes in emotional content of film stimuli. Biological Psychology, 161, 108053. 10.1016/j.biopsycho.2021.108053 33617928

[R49] R Core Team 2016). R: A language and environment for statistical computing [Computer software]. R Foundation for Statistical Computing.

[R50] Reijman S, Alink LRA, Block L. H. C. G. C.d, Werner CD, Maras, Rijnberk C, Ijzendoorn M. H.v, & Bakermans-Kranenburg MJ. (2014). Autonomic reactivity to infant crying in maltreating mothers. Child Maltreatment, 19(2), 101–112. 10.1177/1077559514538115 24879060

[R51] Rodriguez CM, & Richardson MJ. (2007). Stress and anger as contextual factors and preexisting cognitive schemas: Predicting parental child maltreatment risk. Child Maltreatment, 12(4), 325–337. 10.1177/1077559507305993 17954939

[R52] Roos LE, Knight EL, Beauchamp KG, Berkman ET, Faraday K, Hyslop K, & Fisher PA. (2017). Acute stress impairs inhibitory control based on individual differences in parasympathetic nervous system activity. Biological Psychology, 125, 58–63. 10.1016/j.biopsycho.2017.03.004 28268165 PMC5448703

[R53] Roth RM, Isquith PK, & Gioia GA. (2005). BRIEF-A: Behavior rating inventory of executive function - adult version. Psychological Assessment Resources.

[R54] Roth RM, Isquith PK, & Gioia GA. (2014). Assessment of executive functioning using the behavior rating inventory of executive function (BRIEF). In Goldstein S, & Naglieri J (Eds.), Handbook of executive functioning. Springer. 10.1007/978-1-4614-8106-5_18

[R55] Rutherford HJV, Wallace NS, Laurent HK, & Mayes LC. (2015). Emotion regulation in parenthood. Developmental Review, 36, 1–14. 10.1016/j.dr.2014.12.008 26085709 PMC4465117

[R56] Sanders MR, Turner KMT, & Metzler CW. (2019). Applying self-regulation principles in the delivery of parenting interventions. Clinical Child And Family Psychology Review, 22(1), 24–42. 10.1007/s10567-019-00287-z 30788658

[R57] Schulz KP, Fan J, Magidina O, Marks DJ, Hahn B, & Halperin JM. (2007). Does the emotional go/no-go task really measure behavioral inhibition?: Convergence with measures on a non-emotional analog. Archives of Clinical Neuropsychology, 22(2), 151–160. 10.1016/j.acn.2006.12.001 17207962 PMC2562664

[R58] Shahrestani S, Stewart EM, Quintana DS, Hickie IB, & Guastella AJ. (2014). Heart rate variability during social interactions in children with and without psychopathology: A meta-analysis. Journal of Child Psychology and Psychiatry, 55(9), 981–989. 10.1111/jcpp.12226 24635780

[R59] Shahrestani S, Stewart EM, Quintana DS, Hickie IB, & Guastella AJ. (2015). Heart rate variability during adolescent and adult social interactions: A meta-analysis. Biological Psychology, 105, 43–50. 10.1016/j.biopsycho.2014.12.012 25559773

[R60] Skowron EA, Cipriano-Essel E, Benjamin LS, Pincus AL, & Van Ryzin MJ. (2013). Cardiac vagal tone and quality of parenting show concurrent and time-ordered associations that diverge in abusive, neglectful, and non-maltreating mothers. Couple and Family Psychology: Research and Practice, 2(2), 95–115. 10.1037/cfp0000005 24729945 PMC3980485

[R61] Skowron EA, Kozlowski JEM, & Pincus AL. (2010). Differentiation, self-other representations, and rupture-repair processes: Predicting child maltreatment risk. Journal of Counseling Psychology, 57(3), 304–316. 10.1037/a0020030 20729978 PMC2923821

[R62] Smith R, Thayer JF, Khalsa SS, & Lane RD. (2017). The hierarchical basis of neurovisceral integration. Neuroscience & Biobehavioral Reviews, 75, 274–296. 10.1016/j.neubiorev.2017.02.003 28188890

[R63] Smith WH Jr (2003). Emotional cutoff and family stability: Child abuse in family emotional process. In Titelman P (Ed.), Emotional cutoff: Bowen family systems theory perspectives. The Haworth Press, Inc.

[R64] Speidel R, Wang L, Cummings EM, & Valentino K. (2020). Longitudinal pathways of family influence on child self-regulation: The roles of parenting, family expressiveness, and maternal sensitive guidance in the context of child maltreatment. Developmental Psychology, 56(3), 608–622. 10.1037/dev0000782 32077728 PMC7041838

[R65] Steele JS, & Ferrer E. (2011). Latent differential equation modeling of self-regulatory and coregulatory affective processes. Multivariate Behavioral Research, 46(6), 956–984. 10.1080/00273171.2011.625305 26736119

[R66] Stith SM, Liu T, Davies LC, Boykin EL, Alder MC, Harris JM, Som A, McPherson M, & Dees JEMEG. (2009). Risk factors in child maltreatment: A meta-analytic review of the literature. Aggression and Violent Behavior, 14(1), 13–29. 10.1016/j.avb.2006.03.006

[R67] Swain JE, Kim P, Spicer J, Ho SS, Dayton CJ, Elmadih A, & Abel KM. (2014). Approaching the biology of human parental attachment: Brain imaging, oxytocin and coordinated assessments of mothers and fathers. Brain Research, 1580, 78–101. 10.1016/j.brainres.2014.03.007 24637261 PMC4157077

[R68] Tottenham N, Hare TA, & Casey BJ. (2011). Behavioral assessment of emotion discrimination, emotion regulation, and cognitive control in childhood, adolescence, and adulthood. Frontiers in Psychology, 2, 39. 10.3389/fpsyg.2011.00039 21716604 PMC3110936

[R69] U.S. Department of Health and Human Services, Administration for Children and Families, Administration on Children, Youth and Families, Children’s Bureau, Child maltreatment 2020, 2022. https://www.acf.hhs.gov/cb/data-research/child-maltreatment

[R70] van der Put CE, Assink M, Gubbels J, & Boekhout van Solinge NF. (2018). Identifying effective components of child maltreatment interventions: A meta-analysis. Clinical Child and Family Psychology Review, 21(2), 171–202. 10.1007/s10567-017-0250-5 29204796 PMC5899109

[R71] Vila J, Palacios F, Presedo J, Fernández-Delgado M, Felix P, & Barro S. (1997). Time-frequency analysis of heart-rate variability. IEEE Engineering in Medicine and Biology Magazine, 16(5), 119–126. 10.1109/51.620503 9313089

[R72] Wells JN, Skowron EA, Scholtes CM, & Degarmo DS. (2020). Differential physiological sensitivity to child compliance behaviors in abusing, neglectful, and non-maltreating mothers. Development and Psychopathology, 32(2), 531–543. 10.1017/s0954579419000270 31060634 PMC7607912

[R73] Zhang X, Gatzke-Kopp LM, Cole PM, & Ram N. (2022). A dynamic systems account of parental self-regulation processes in the context of challenging child behavior. Child Development, 93(5), 501–514. 10.1111/cdev.13808 35635069

[R74] Zimmerman PH, Bolhuis JE, Willemsen A, Meyer ES, & Noldus LPJJ. (2009). The Observer XT: A tool for the integration and synchronization of multimodal signals. Behavior Research Methods, 41(3), 731–735. 10.3758/brm.41.3.731 19587185

