# Supplementary material for: Dynamic regulatory processes among child welfare parents: Temporal associations between physiology and parenting behavior
Source: Dev Psychopathol. Author manuscript; Available in PMC 2025 Feb 7. (PMC10847384; doi:10.1017/S0954579423000949)
Supplement: 1 [file NIHMS1916126-supplement-1.pdf]

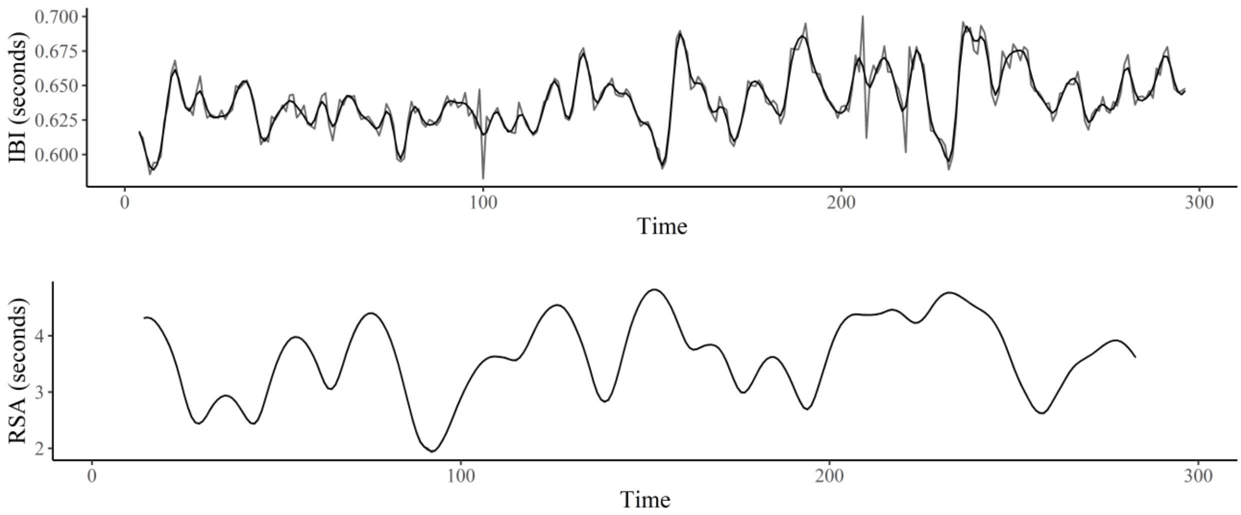

Figure S1. Visualization of raw and smoothed time-series data of one parent's IBI and RSA estimates during the child-led play task

Note. Because the moving-window approach in estimating dynamic changes in RSA has essentially smoothed the time-series output already, minimal smoothing was done further ($\lambda = 0.1$) in the first step of ordinary equation modeling. Thus, the raw and smoothed RSA data almost fully overlapped in the figure. Time-series estimates of IBI were smoothed with $\lambda = 0.5$.

Table S1. *Parameter Estimates for the Models Examining Intra-Individual Dynamic Associations Between IBI and Positive Parenting Behaviors*

Fixed Effect	Estimate (*SE*)	*p*
Predicting momentary changes in IBI		
γ_{a10} (Intercept of IBI changes)	3.40E-5 (0.0001)	.76
γ_{b10} (Momentary level of IBI)	0.0007 (0.0017)	.67
γ_{c10} (Local density of positive behavior)	-0.0014 (0.0005)	.007
Predicting momentary changes in positive behavior		
γ_{d10} (Intercept of positive behavior changes)	4.12E-5 (0.0005)	.93
γ_{e10} (Local density of positive behavior)	0.0013 (0.0015)	.37
γ_{f10} (Momentary level of IBI)	0.0338 (0.0117)	.004
**Random Effect**	**Estimate**	**95% *CI***
Standard deviation		
$\sigma_{w_{a1i}}$	0.0004	[0.0002, 0.0008]
$\sigma_{w_{c1i}}$	0.0046	[0.0037, 0.0057]
$\sigma_{w_{d1i}}$	-	-
$\sigma_{w_{f1i}}$	0.1119	[0.0891, 0.1406]
σ_{u1i} (Residual of IBI changes)	0.0247	[0.0245, 0.0248]
σ_{v1i} (Residual of positive behavior changes)	0.1052	[0.1046, 0.1058]

Note. IBI = Inter-beat interval; *SE* = Standard error; *CI* = Confidence interval. Model estimates suggested that the random effect for the intercept of positive behavior changes (w_{d1i}; the expected momentary change in positive parenting behaviors when parents were showing no positive behaviors and task-average level of IBI) was minimal, so it was set to 0 in the final model.

Table S2. *Parameter Estimates for the Models Examining Intra-Individual Dynamic Associations Between IBI and Negative Parenting Behaviors*

Fixed Effect	Estimate (*SE*)	*p*
Predicting momentary changes in IBI		
γ_{a20} (Intercept of IBI changes)	3.44E-5 (0.0001)	.80
γ_{b20} (Momentary level of IBI)	0.0002 (0.0017)	.91
γ_{c20} (Local density of negative behavior)	-0.0005 (0.0002)	.06
Predicting momentary changes in negative behavior		
γ_{d20} (Intercept of negative behavior changes)	-0.0002 (0.0010)	.87
γ_{e20} (Local density of negative behavior)	0.0006 (0.0015)	.66
γ_{f20} (Momentary level of IBI)	0.0615 (0.0261)	.02
**Random Effect**	**Estimate**	**95% *CI***
Standard deviation		
$\sigma_{w_{a2i}}$	0.0008	[0.0006, 0.0011]
$\sigma_{w_{c2i}}$	0.0023	[0.0018, 0.0028]
$\sigma_{w_{d2i}}$	1.1791E-6	[3.2973E-13, 4.2167]
$\sigma_{w_{f2i}}$	0.2719	[0.2248, 0.3288]
σ_{u2i} (Residual of IBI changes)	0.0247	[0.0245, 0.0248]
σ_{v2i} (Residual of negative behavior changes)	0.2078	[0.2066, 0.2090]

Note. IBI = Inter-beat interval; *SE* = Standard error; *CI* = Confidence interval.

Table S3. *Parameter Estimates for the Models Examining Intra-Individual Dynamic Associations Between RSA and Positive Parenting Behaviors*

Fixed Effect	Estimate (*SE*)	*p*
Predicting momentary changes in RSA		
γ_{a30} (Intercept of RSA changes)	-0.0005 (0.0005)	.39
γ_{b30} (Momentary level of RSA)	0.0001 (0.0006)	.87
γ_{c30} (Local density of positive behavior)	0.0007 (0.0046)	.88
Predicting momentary changes in positive behavior		
γ_{d30} (Intercept of positive behavior changes)	-0.0001 (0.0005)	.82
γ_{e30} (Local density level of positive behavior)	-0.0013 (0.0016)	.42
γ_{f30} (Momentary level of RSA)	-0.0008 (0.0007)	.25
**Random Effect**	**Estimate**	**95% *CI***
Standard deviation		
$\sigma_{w_{a3i}}$	0.0039	[0.0030, 0.0051]
$\sigma_{w_{c3i}}$	0.0573	[0.0502, 0.0654]
$\sigma_{w_{d3i}}$	-	-
$\sigma_{w_{f3i}}$	0.0031	[0.0013, 0.0077]
σ_{u3i} (Residual of RSA changes)	0.0996	[0.0990, 0.1002]
σ_{v3i} (Residual of positive behavior changes)	0.1053	[0.1047, 0.1060]

Note. RSA = Respiratory sinus arrhythmia; *SE* = Standard error; *CI* = Confidence interval. Model estimates suggested that the random effect for the intercept of positive behavior changes (w_{d3i}; the expected momentary change in positive parenting behaviors when parents were showing no positive behaviors and task-average level of RSA) was minimal, so it was set to 0 in the final model.

Table S4. *Parameter Estimates for the Models Examining Intra-Individual Dynamic Associations Between RSA and Negative Parenting Behaviors*

Fixed Effect	Estimate (*SE*)	*p*
Predicting momentary changes in RSA		
γ_{a40} (Intercept of RSA changes)	0.0004 (0.0007)	.62
γ_{b40} (Momentary level of RSA)	0.0002 (0.0006)	.72
γ_{c40} (Local density of negative behavior)	-0.0006 (0.0017)	.73
Predicting momentary changes in negative behavior		
γ_{d40} (Intercept of negative behavior changes)	-0.0002 (0.0011)	.86
γ_{e40} (Local density of negative behavior)	-0.0009 (0.0015)	.58
γ_{f40} (Momentary level of RSA)	0.0010 (0.0012)	.39
**Random Effect**	**Estimate**	**95% *CI***
Standard deviation		
$\sigma_{w_{a4i}}$	0.0070	[0.0058, 0.0084]
$\sigma_{w_{c4i}}$	0.0212	[0.0186, 0.0242]
$\sigma_{w_{d4i}}$	-	-
$\sigma_{w_{f4i}}$	-	-
σ_{u4i} (Residual of RSA changes)	0.0999	[0.0993, 0.1005]
σ_{v4i} (Residual of negative behavior changes)	0.2080	[0.2068, 0.2093]

Note. RSA = Respiratory sinus arrhythmia; *SE* = Standard error; *CI* = Confidence interval. Model estimates suggested that the random effects for the intercept of negative behavior changes and how changes in negative behaviors were predicted by the momentary level of RSA (w_{d4i} and w_{f4i}) were minimal, suggesting little inter-individual differences in those intra-individual parameters; thus, those random effects were set to 0, which removed the nested modeling structure for that specific model.

Table S5. *Post hoc analyses examining parents' self-reported affirmative parenting behaviors as a moderator for physiology-positive behavior dynamic associations*

Description of the *Parental Affirmation* measure	Parent completed the Structural Analysis of Social Behavior Intrex Questionnaires – Short Form, a self-report assessment of intra- and inter-personal perceptions and characteristics (Benjamin, Rothweiler, & Critchfield, 2006). One item from the Me with My Child – Transitive Scale (Clusters 12 – parent affirms child) capturing parents' effort to respond to their children in an affirming and understanding way was rated on a continuous scale from 0 (does not apply at all/never) to 100 (applies perfectly/all the time).
Descriptive statistics	On a possible range of 0 to 100, scores in this sample ranged widely from 30 to 100, although the sample mean was closer to the higher end of the scale, *M* (*SD*) = 90.69 (12.76). Scores were standardized based on sample mean and standard deviation before entered into the models to predict the dynamic associations between parental IBI and observed positive behaviors during the child-led play. Parents' self-reported affirmation was not correlated with their self-reported harsh attribution about child behaviors (r = -.03, p = .72).
Findings	Parents' self-reported affirmative parenting behaviors in daily life significantly moderated how observed positive behaviors predicted momentary changes in IBI (coefficient of the interaction effect = 0.0019, *SE* = 0.0005, p < .001), as well as how IBI level predicted changes in positive behaviors (coefficient of the interaction effect = -0.0253, *SE* = 0.0114, p = .03) during the child-led play task. The negative feedback loop, where positive behaviors predicted increases in cardiac arousal, which in turn was related to decreases in positive behaviors, was only evident among parents reporting average or lower levels (*M* - *SD*) of affirmative behaviors toward their children, but not among those reporting higher levels of affirmative behaviors (*M* + *SD*).<br><br>On the contrary, parents reporting higher levels of affirmative behaviors demonstrated associations between RSA and positive parenting dynamics. Self-reported affirmative parenting was a significant moderator of both how observed positive behaviors predicted momentary changes in RSA (coefficient of the interaction effect = 0.0124, *SE* = 0.0047, p = .008) and how RSA level predicted changes in positive behaviors (coefficient of the interaction effect = -0.0018, *SE* = 0.0006, p = .002). For parents reporting more affirmative parenting behaviors (*M* + *SD*), their positive behaviors tended to increase at moments of lower RSA, and more positive behaviors were in turn related to a momentary increase in RSA. Such patterns were not evident among parents reporting average or lower levels (*M* - *SD*) of affirmative parenting who showed IBI-positive behavior dynamic associations.
